# Supplementary material for: Genomic portrait and relatedness patterns of the Iron Age Log Coffin culture in northwestern Thailand
Source: Nat Commun. 2023 Dec 22;14:8527. doi: 10.1038/s41467-023-44328-2 (PMC10746721; doi:10.1038/s41467-023-44328-2)
Supplement: Supplementary file 1 — Supplementary Information [file 41467_2023_44328_MOESM1_ESM.pdf]

Supplementary Information for:

Genomic portrait and relatedness patterns of the Iron Age Log Coffin culture in northwestern Thailand

Selina Carlhoff[1*], Wibhu Kutanan[2], Adam B Rohrlach[1,3], Cosimo Posth[4,5], Mark Stoneking[6,7], Kathrin Nägele[1], Rasmi Shoocongdej[8,9*], Johannes Krause[1*]

[1]Department of Archaeogenetics, Max Planck Institute for Evolutionary Anthropology, Leipzig, Germany.

[2]Department of Biology, Faculty of Science, Naresuan University, Phitsanulok, Thailand.

[3]School of Computer and Mathematical Sciences, University of Adelaide, Adelaide, Australia.

[4]Institute for Archaeological Sciences, Archaeo- and Palaeogenetics, University of Tübingen, Tübingen, Germany.

[5]Senckenberg Centre for Human Evolution and Palaeoenvironment, University of Tübingen, Tübingen, Germany.

[6]Department of Evolutionary Genetics, Max Planck Institute for Evolutionary Anthropology, Leipzig, Germany.

[7]Université Lyon 1, CNRS, Laboratoire de Biométrie et Biologie Evolutive, UMR 5558, Villeurbanne, France.

[8]Department of Archaeology, Silpakorn University, Bangkok, Thailand.

[9]The Prehistoric Population and Cultural Dynamics in Highland Pang Mapha Project, Princess Maha Chakri Sirindhorn Anthropology Centre, Bangkok, Thailand.

*Corresponding authors: selina_carlhoff@eva.mpg.de, SHOOCONGDEJ_R@su.ac.th, krause@eva.mpg.de

Supplementary Notes

Site descriptions

Ban Rai (BRB)

Ban Rai (19° 31'18" N 98° 10'31" E) is a rock shelter on top of the southern edge of the Lang River valley near Ban Huai Rai (Ban Hai) village in the Pang Mapha District of Mae Hong Son Province. Excavations under the Highland Archaeology Project led by Rasmi Shoocongdej yielded a rich, well-dated stratigraphic sequence indicating two occupation layers and prehistoric rock paintings of human figures and animals[1–9]. The pre-Log Coffin culture layers have been dated to 12.5 to 8 k BP and were characterized by a wide range of lithics[10,11], an abundance of faunal remains[12], and a primary flexed burial[1].

In its second phase, the rock shelter was used exclusively as a burial site assigned to the Log Coffin culture (2040±30-1770±30 BP)[13]. This layer yielded human remains, pottery sherds, iron tools (chisel, hoop), and 15 log coffins and supporting posts dug into older layers[1,9]. Dendrochronological and ^{14}C studies of log coffin heads showed the simultaneous use of different carving styles[14,15], while oxygen chronology indicated that precipitation was higher during the Log Coffin period[16].

Lahu Pot (LHP)/Moa Mussur (MMS)

Lahu Pot (19° 35' N, 98° 15' E) is a stalagmite cave located in the Lang River basin near Wa-Na Luang village in the Pang Mapha District. The lower cave yielded six log coffins, human bones, and teeth, while four log coffins and human bones were found in the upper cave. Dates from Lahu Pot Cave range from 1,540 ± 120 to 2,080 ± 60 BP[17].

Pang Kham 1 (PKL)

Pang Kham 1 Cave (19° 40' N, 98° 12' E) is located 1,034 m above sea level in the Huay Pong Saen Pig basin in the Pang Mapha District. It can be divided into three areas of which only two housed log coffins. The second area yielded several log coffins, iron tools, bronze decorations, earthenware, and human bones. The third area housed many coffins, earthenware, and a limestone-embedded skull. A total of 10 log coffins were recovered and dated to 1,343 ± 59 BP and 1,698 ± 59 BP[18].

Tham Lod rock shelter (THL)

The Tham Lod rock shelter (19° 34' N 98° 16' E) is located at the base of a limestone cliff overhang around 250 m from a Nam Lang River tributary in the vicinity of the Tham Lod Natural and Wildlife Education Center. It was excavated from 2002 to 2006 under the direction of Rasmi Shoocondej (Highland Archeological Project in Pang Mapha) supported by Thailand Research Funds (TRF) and Silpakorn University[2–9].

Tham Lod yielded the longest stratigraphic sequence of the region (10 layers over 4.5 m). More than 100,000 archaeological remains date from the late Pleistocene into the Holocene, including lithic artefacts, faunal and floral remains, pottery sherds, and metal objects from upper layers[7,12]. Dates produced from various materials indicate use as a temporary settlement for lithic production[10,11,19], including Hòabìnhian-typical stone artefacts (sumatraliths) in layers 4 to 6[20], food preparation, and as a cemetery[8,9,21] from 35,782 ± 266 BP (layer 10) over 27,620 ± 170 BP (layer 8) to ~3 kaa ago (layer 2), albeit the absence of the Middle Holocene[20]. Recent isotopic studies of human and faunal teeth enamel tracked the expansion of forest-grassland ecosystems in the region. They suggested a northern expansion of the savanna corridor during the terminal Pleistocene providing favourable conditions for hunter-gatherers across mountainous areas[22].

Two burials and two fragmentary skeletons were discovered in the youngest Pleistocene layers associated with faunal remains, shells, flakes, and used cobble tools[8,23,24].

Tham Lod cave (THL)

Tham Lod cave (19° 34' N, 98° 17' E) is situated near the Tham Lod Natural and Wildlife Education Center, about 300 m from the Tham Lod rockshelter. The cave is a multi-chamber cave complex with the Nam Lang river passing through the cave. The burial site is located in the upper chambers, where three chambers contain fragments of ten log coffins, earthenware sherds, iron rings, and a decorated tooth, but the site has been badly disturbed by humans and animals. Pieces of log coffin from the site date to 1,450±110 - 1240±90 BP[17].

Ban Wang Hai (WAH)

Ban Wang Hai (18° 32' N, 99° 00' E) is situated in Kuang river valley, Mueang Lamphun district, Lamphun province. The site was first excavated by the 4th Regional Office of Fine Arts Department in 1987. Seven skeletal remains were discovered along with grave goods including bronze and glass bracelets, iron tools, stone tools, spindle whorls and earthenware pottery as well as stone and glass beads (the 4th Regional Office of Fine Arts Department

1987). The excavation was continued as part of the Thai-French Prehistoric Project between 1996-1998 and dated to 429-657 CE[25].

Yappa Nhae 1 (YPN)

Yappa Nhae 1 Cave (19° 36' N, 98° 14' E) is located 900 m above sea level in the Mae La Na Basin near Ya-Pa Nae village in the Pang Mapha District. Indirectly dated between 2,200 and 1,200 BP, four log coffins were found in Chamber 3 and 4 in addition to human remains of at least nine individuals (five adults, two juveniles, one baby, one unknown), a decorated tooth, iron tools, bronze coins, and earthenware.

Yappa Nhae 2 (YPN)

Yappa Nhae 2 Cave (19° 36' N, 98° 14' E) is located 964 m above sea level in the Mae La Na basin near Ya-Pa Nae village in the Pang Mapha District. Four log coffins were discovered in the middle, upper and lower chamber indirectly dated to 2,020 to 1,200 BP from the log coffin heads[26]. These were associated with human remains of at least 23 individuals (19 adults and four children), earthenware, chisels, animal bones, and iron tools.

Supplementary Methods

<u>Shotgun screening</u>

After demultiplexing, the shotgun-sequencing data was processed with nf-core/eager v2.4.0 (https://nf-co.re/eager)[27] using Nextflow v21.04.0[28]. General sequencing quality control was conducted with FastQC v0.11.9[29] and fastP v0.20.1[30]. Forward and reverse adapters were removed using AdapterRemoval v2.3.2[31] with one overlapping position trimming reads based on 30 bp read length and 20 base quality and skipping base quality trimming on the 5' end. Using BWA v0.7.17-r1188[32,33], the reads were aligned to the human reference genome *hg19* allowing for 1 mismatch per read, maximum edit distance of 2, disabling the seeding and two gaps. The resulting bam files were then filtered with SAMtools v1.12[34] for a minimum mapping quality and minimum read length of 30, and unmapped reads were discarded. Mapping statistics were generated with Qualimap v2.2.2-dev[35] and bedtools v2.30.0[36]. Duplicates were removed using Picard MarkDuplicates v2.26.0 (http://broadinstitute.github.io/picard/) and C->T misincorporation frequency assessed with DamageProfiler v0.4.9[37] of all reads up to 100 bp, considering 15 bp of each read and setting the maximum misincorporation frequency to be displayed. Endogenous DNA was estimated using endorS.py v0.4 (https://github.com/aidaanva/endorS.py), and the final report generated with MultiQC v1.11[38].

```
nextflow run nf-core/eager
        -profile eva,archgen,big_data
        -r 2.4.0
        --input Thailand.tsv
        --min_adap_overlap 1
        --clip_readlength 30
        --clip_min_read_quality 20
        --preserve5p
        --mapper bwaaln
        --bwaalnnn 0.01
        --bwaalno 2
        --run_bam_filtering true
        --bam_mapping_quality_threshold 30
        --bam_filter_minreadlength 30
        --bam_unmapped_type discard
        --dedupper markduplicates
        --damageprofiler_length 100
        --damageprofiler_threshold 15
        --damageprofiler_yaxis 0.3
```

In-solution enrichment

The 1240K-enriched sequences were processed with nf-core/eager v2.4.0 as described above with the addition of providing the 1240K positions in a BED file, mitochondrial to nuclear ratio calculation using MTNucRatioCalculator v0.7, genetic sex estimation with sexdeterrmine v1.1.2[39] and nuclear contamination estimation with ANGSD v0.935[40]. After trimming off 2 bp at both ends of the reads with bamUtil v1.0.15[41], the resulting bam files were genotyped with pileupCaller from sequenceTools v1.4.0.5 (https://github.com/stschiff/sequenceTools) on the 1240K SNP panel calling random haploids and utilizing all sites.

```
nextflow run nf-core/eager
        -profile eva,archgen,big_data
        -r 2.4.0
        --input Thailand.tsv
        --min_adap_overlap 1
        --clip_readlength 0
        --clip_min_read_quality 20
        --preserve5p
        --mapper bwaaln
        --bwaalnnn 0.01
        --bwaalno 2
        --run_bam_filtering true
        --bam_mapping_quality_threshold 30
        --bam_filter_minreadlength 30
        --bam_unmapped_type discard
        --dedupper markduplicates
        --damageprofiler_length 100
        --damageprofiler_threshold 15
        --damageprofiler_yaxis 0.3
        --snpcature_bed
        --run_mtnucratio true
        --mtnucratio_header MT
        --run_sextederrmine true
        --run_nuclear_contamination true
        --contamination_chrom_name chrX
        --run_trim_bam true
        --bamutils_clip_half_udg_left 2
        --bamutils_clip_half_udg_right 2
        --run_genotyping true
        --genotyping_tool pileupcaller
        --genotyping_source trimmed
        --pileupcaller_method randomHaploid
        --pileupcaller_transition AllSites
```

Supplementary Discussion

Ancient DNA authentication

After shotgun sequencing, the level of endogenous human DNA content ranged from <0.1% to 57.92%, with a median of 0.285% (Supplementary Data 1). Overall, samples from Yappa Nhae were better preserved compared to the other sites, and petrous bones yielded much more endogenous DNA compared to teeth.

We assessed if the sequenced reads were ancient rather than present-day human DNA by the rate of cytosine to thymine mis-incorporations at the 5' end of reads ('ancient DNA damage', Supplementary Figure 2A, Supplementary Data 1). Most samples showed an elevated damage rate (7-26%) at the first base, which drops off further into the read due to UDG-half treatment. Since ancient DNA reads are known to be fragmented, we also evaluated the median read length, which ranged between 30 and 55 bp (Supplementary Figure 2B, Supplementary Data 1). Based on previous comparisons of ancient and present-day DNA[42], 33 sequences showed the damage pattern and read length characteristic of ancient DNA and an endogenous DNA content suitable for in-solution capture (>0.1%).

Runs of homozygosity

We also used hapROH v0.6[43] to estimate effective population size (N_e), i.e., the size of an idealized population that would give rise to the same variance of allele frequencies, with a maximum likelihood approach (MLE_ROH_Ne) to 977 (845-1,130) after removing the four individuals with ROH >20 cM. For the individual sites, we calculated the following N_e values: 50,000 (2,610-50,000) for Ban Rai, 1,047 (483-2,936) for Lahu Pot, 1,316 (784-2,453) for Tham Lod, and 1,047 (881-1,254) for Yappa Nhae and 518 (382-716) for Long Long Rak.

IBD calculation

To further investigate the genetic relationships between the studied individuals, we analysed long shared segments of the genome, also called identity-by-descent (IBD) blocks. Due to sequencing differences and low coverage, we were not able to include the published individuals from Long Long Rak in our analysis[44]. To assess imputation accuracy, we also plotted genotypes before and after imputation onto a PCA calculated on present-day East and Southeast Asian groups, as described previously. In PCA space, the imputed individuals are

more spread out and show a slight drift towards groups from East Asia, but generally overlap with the positions of the non-imputed individuals (Supplementary Figure 5A).

Principal component analysis

We merged the newly generated genotype calls with published data from present-day and ancient individuals from northern to southern East Asia[44–55] and used smartpca v16000 (lsqmode: YES, shrinkmode: YES) from EIGENSOFT v7.2.1[56] to calculate several sets of principal components.

The initial PCA was calculated on present-day East and Southeast Asian individuals[45,46,48,49,53,55] and with all ancient individuals projected onto the scaffold from the present-day individuals (Supplementary Figure 6A). Along PC1 we can observe a north-south cline, while PC2 stretches between the Onge from the Andaman Islands and the Taiwanese Atayal. The newly genotyped ancient individuals cluster together with present-day individuals from Thailand (Padaung Karen, Eastern Lawa, Western Lawa, Mon) as well as the Log Coffin-associated individuals from Long Long Rak[49]. This position is markedly different from Bronze and Iron Age individuals from Ban Chiang, northeastern Thailand[54] and other contemporaneous individuals from Guangxi, China[53].

The second PCA was calculated on the present-day individuals from Thailand only[49] which separated according to the genetic drift of specific groups (Supplementary Figure 6B). The ancient individuals clustered with present-day groups affiliated with all language families except Hmong-Mien (Blang, Padaung Karen, Laotian, Eastern Lawa, Western Lawa). However, the Ban Chiang individuals were only associated with Austroasiatic-speaking groups, such as the Bru, Khmu, and Soa, and the younger individuals from Guangxi clustered with Hmong-Mien speakers.

f-statistics

To formally investigate the observed patterns in the PCA and ADMIXTURE analyses, we used qp3Pop v651 and qpDstat v980 from AdmixTools v7.0.2[45].

First, we tested the different affinities of the Ban Chiang Bronze Age individuals[49] and the ancient individuals from northwestern Thailand to all published ancient groups from East Asia[49] with f_4(Mbuti.DG, ancient East Asia; Thailand_BA, Thailand_1700BP). While there

were no ancient groups that showed closer affinity to the Bronze Age individuals, ancient individuals from northern East Asia (e.g., Miaozigou_MN, Upper_YR_IA, WLR_LN, Mongolia_EIA_7_Xiognu, Upper_YR_LN, YR_LBIA, Mongolia_EIA_3, Mongolia_LBA_2_Ulaanzukh) and Nepal (Mebrak_2125BP.SG, Samdzong_1500BP.SG) resulted in significantly positive values indicating extra genetic affinity present in the ancient individuals from northwestern Thailand (Supplementary Figure 7A).
We also observed this pattern when comparing with the Iron Age individual from Ban Chiang[55], albeit with a lower number of overlapping SNPs.

Tests with Long Long Rak in the third position showed significant attraction of the ancient individuals from Thailand to Neolithic individuals from Vietnam, Tanshishan, Mongolia_EIA_8, Bianbian, and Boisman_MN, while Vietnam_BA_DongSonCulture.SG was significantly closer to Long Long Rak (Supplementary Figure 7B).

The statistic f_3(Mbuti; present-day groups, Thailand 1700BP) indicated that the ancient individuals from northwestern Thailand shared most drift with the Austroasiatic-speaking Mlabri and Htin from Thailand[53] followed by the Kra-Dai-speaking Maonan and Gelao from southern China[44] (Supplementary Figure 8). The tested groups also included present-day groups from Thailand[53], Malaysia[49] and other South and East Asian groups[45,46].

The results of f_4(Mbuti, present-day Thai; Bronze Age Ban Chiang, Thailand 1700BP) showed a closer affinity of all present-day groups from Thailand[57] to the newly genotyped ancient individuals compared to the Bronze Age individuals from northeastern Thailand[49]. The values were particularly high for the Palaung, Skaw Karen, Iu Mien, and Shan, whereas tests including the Khmu, Kalueang, Soa, Austronesian speakers from southern Thailand, Htin Pray and Bru were not significant (Supplementary Figure 9).

We also tested if any present-day groups from Thailand showed closer affinity to the Log Coffin-associated genomes compared to other present-day groups (f_4(Mbuti,present-day Thailand; Thailand1700BP, present-day groups). Here, we were particularly interested in the hill tribes living in the highlands (Karen, Lahu, Lisu, Akha, Lawa, Htin, Khmu, Hmong, and Iu Mien), who are culturally and genetically isolated from the lowlanders[44], and chose the Sino-Tibetan-speaking Padaung Karen, the Austroasiatic-speaking Htin Pray and the Hmong Mien-speaking Hmong Njua for statistical testing based on close geographical or position in PCA space. Since Tai-Kadai languages have been hypothesized to have spread from southern

China in the Iron Age[54], we repeated the same test with the Tai-Kadai speaking Shan in the second position, who live near the excavation site.

The results indicate that the Log Coffin-associated individuals are closer to the tested groups when compared with the Onge, Jehai, and Salar, while the tested groups are all genetically closer to other groups of the same language family than to the ancient genomes (Supplementary Figure 10). Since there is no present-day group or groups from one language family, which show a closer genetic affinity to the ancient genomes, we conclude that the present-day genetic diversity in Thailand is the result of different demographic processes than captured by the ancient genomes from northwestern Thailand.

qpAdm

We then used the f_4 results to inform ancestry modelling with qpAdm v1520 from AdmixTools v7.0.2[45].

We first tried to model the ancient individuals from Thailand as an admixture between Bronze Age Ban Chiang and ancient individuals from northern East Asia, which showed the strongest affinity to the Log Coffin-associated individuals (Miaozigou_MN, Upper_YR_IA, WLR_LN, Mongolia_EIA_7_Xiognu, Upper_YR_LN, YR_LBIA, Mongolia_EIA_3, Mongolia_LBA_2_Ulaanzukh, Mebrak_2125BP.SG, Samdzong_1500BP.SG, Fig. S8). The published ancient individuals were rotated from left (sources) to right (references). The tested groups from the Yellow River region resulted in well-fit models estimating around 50% northern East Asian ancestry in the ancient individuals from northwestern Thailand (Supplementary Figure 11).

A parallel setup modelling the ancestry of the Long Long Rak individuals resulted in very similar results. However, the amount of Bronze Age Ban Chiang ancestry was slightly higher in all models.

Setting the Iron Age individuals from Ban Chiang as the target, however, showed that no genetic input from for northern East Asian groups was required (nested $p>0.05$).

Initially it was suggested that all Neolithic Southeast Asian individuals, including the individuals from Ban Chiang, represent an admixture between deep Onge- (hunter-gatherer) and Ami-related (early farmer) groups[44], where Hòabìnhian-associated individuals from Laos and Malaysia were the best proxies for the hunter-gatherer ancestry[54]. However, later studies showed that another hunter-gatherer source, Longlin from Guangxi Province[52], produced

better fitting models for Neolithic Man Bac in Vietnam and some other ancient Southeast Asians, while others retained Hòabìnhian-associated influence[44,49,52,54]. To account for this diverse ancestry set present in the region and assess the predominant genetic influence for all ancient groups from Thailand, we tested models using both the Hòabìnhian-associated individual from Laos[58] and the Longlin individual from Guangxi[44] as the hunter-gatherer source or in the reference groups. As representatives for early farmer ancestry we rotated the Neolithic individuals from southern China[49] from sources to references.

For the newly genotyped individuals using the Hòabìnhian-associated individual as the hunter-gatherer source resulted in better fitting models, i.e., higher p-values, although most models with the Longlin individual did fit ($p>0.05$). The best-fitting model estimated 36.0 ± 3.1% Hòabìnhian-related and 64.0 ± 3.1% Suogang-related ancestry (Supplementary Figure 12).

The Hòabìnhian-associated individual was also the preferred source for Long Long Rak with the best-fitting model favouring Liangdao1 as the southern East Asian source.

The models for the Iron Age individuals from Ban Chiang resulted in larger hunter-gatherer ancestry components compared to the Iron Age groups from northwestern Thailand and the differentiation between models including the Hòabìnhian and Longlin as sources was less pronounced.

Bronze Age Ban Chiang was best modelled as an admixture between Longlin and Qihe. Overall, the best-performing southern East Asian source varied, but most models involving Liangdao1 showed sufficient model-fit, and hence was selected for further modelling.

Combining the information from the previous two analyses, we simultaneously estimated ancestry proportions related to hunter-gatherers, early farmers, and northern East Asians. We set the Hòabìnhian-associated individual from Laos and Liangdao1 as sources, while the remaining southern East Asian individuals were added to the references, and all northern East Asians rotated from references to sources (Supplementary Figure 13, Supplementary Data 5). The model could not assign any ancestry related to northern East Asia in the Bronze and Iron Age individuals from Ban Chiang, whereas the new individuals from northwestern Thailand showed a large component related to Upper_YR_LN, Miaozigou_MN, and Upper_YR_IA. However, using this setup, we could not find a sufficiently well-fit model for Long Long Rak ($p<0.05$).

To investigate the geographical spread of the northern East Asian-related ancestry, we attempted to fit most previously published individuals as an admixture between a Longlin/Hòabìnhian-associated individual, Liangdao1 and northern East Asian[50]. While we could model Laos_Unknown.SG, Thailand 1700BP, Vietnam_Historical.SG and Vietnam_LN.SG as Longlin, Liangdao1 and Upper Yellow River, the models with the Hòabìnhian-associated individual achieved higher p-values and fit for more northern East Asian groups. Our analyses indicate that hunter-gatherer-related ancestry is not necessary for the 1.5-0.5-ka-old individuals from Guangxi (BaBanQinCen, GaoHuaHua, LaCen, Shenxian and Yiyang), Myanmar_LN_BA_lc from Oakaie and Vietnam_BA from Nui Nap (nested model 100 $p>0.05$ or negative admixture proportion). Conversely, northern East Asian ancestry is not required for modelling Baojianshan, Dushan, Thailand_BA, Thailand_IA (Ban Chiang) and Vietnam_N (Man Bac) (nested model $p>0.05$).

We repeated the analysis described above for the newly sequenced genomes individually using the Hòabìnhian-associated individual, Liangdao1, and Upper YR LN as respective proxies with the remaining ancient genomes as references (Mbuti.DG, Longlin, Qihe, Xitoucun, Tanshishan, Suogang, Miaozigou_MN, Mongolia_EIA_3, Mongolia_EIA_7_Xiongnu, Mongolia_LBA_2_Ulaanzukh, Nepal_Mebrak_2125BP.SG, Nepal_Samdzong_1500BP.SG, Upper_YR_IA, WLR_LN, YR_LBIA). We could show some variation in hunter-gatherer, early farmer, and northern East Asian ancestry, but overall, the proportions were similar, around 15%, 45%, and 40%, respectively (Supplementary Figure 14). The lowest northern East Asian component was estimated for THL003, while THL004 could also be modelled without any hunter-gatherer contribution (nested $p>0.05$). This ancestry model did not fit for YPN010, YPN017, and YPN030 ($p<0.05$).

<u>ADMIXTURE</u>

We estimated proportions of ancestry using *ADMIXTURE* v.1.3.0[59] on a dataset containing present-day and ancient individuals from East and Southeast Asia[44–46,49,50,52–54,59]. For K=8 the corresponding ancestry components are maximized in the following groups: Mbuti (pink), French (light blue), Onge (dark green), Mlabri (dark purple), Atayal (red), Lahu (light green), Htin Pray (yellow), Boisman_MN (orange; Supplementary Figure 16). The newly generated genomes from northwestern Thailand are split into ~65% Boisman and ~35% Htin Pray ancestry component with most individuals additionally containing a few per cent Mlabri, and some individuals very little French or Atayal ancestry. This is similar to the individuals from

Long Long Rak (Thailand_IA.SG/WGC)[49], but differs from Ban Chiang (Thailand_BA)[60], which lack the Boisman and contain more Lahu component.

Sex-biased admixture

Due to the lack of X-chromosome coverage in individuals from northern East Asia[43], we were not able to conduct the qpAdm analyses exclusively on X-chromosome data to assess differences in admixture proportions on the autosome and X-chromosome potentially related to sex-biased admixture.

An ADMIXTURE analysis including present-day individuals from Thailand was similarly inconclusive.

Admixture time dating

We used DATES v753[60] to estimate the time of admixture between the hunter-gatherer/Yangtze River, hunter-gatherer/Yellow River and Yangtze River/Yellow River components. However, the linkage disequilibrium decay curves were flat and the resulting standard errors very large, when using Laos_Hoabinhian, Liangdao1 and Upper_YR_LN as representatives for these ancestry components.

Supplementary Figures

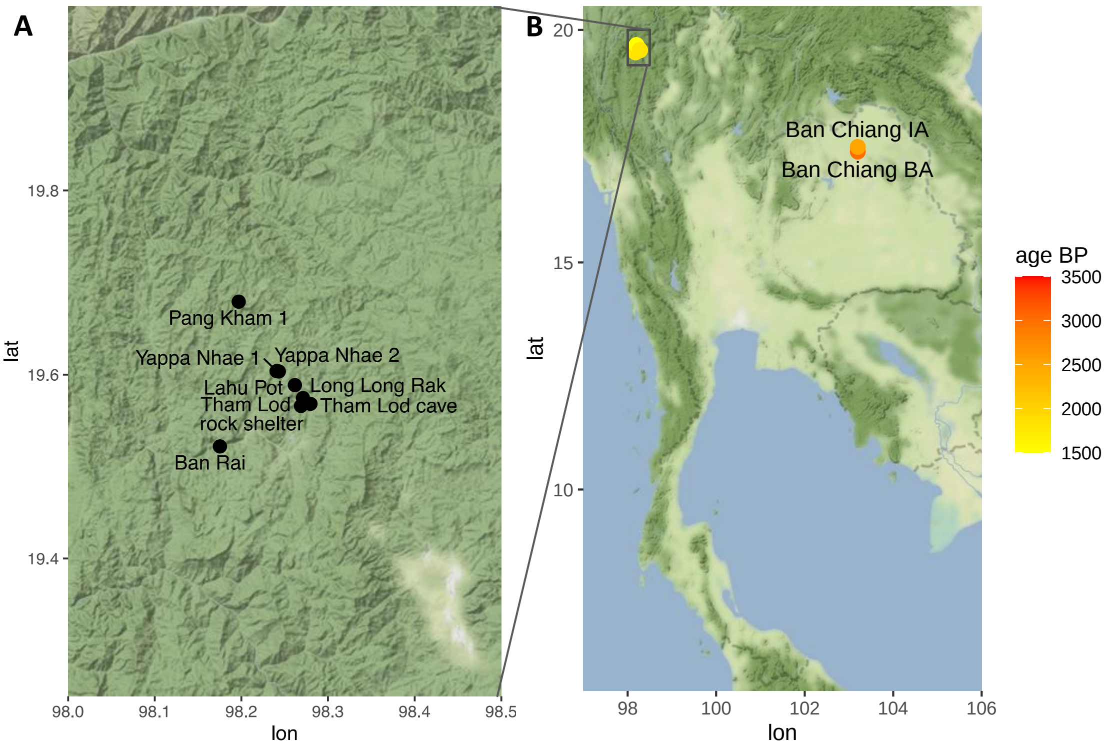

Supplementary Figure 1. Maps of archaeological sites used in this study; (**A**) the location of the archaeological sites from which data was produced for this study and Long Long Rak[53]; (**B**) the location of archaeological sites in Thailand for which ancient DNA data has been published[49]; map created with ggmap v3.0.0[61], map tiles by Stamen Design (CC BY 3.0), data by OpenStreetMap (ODbL).

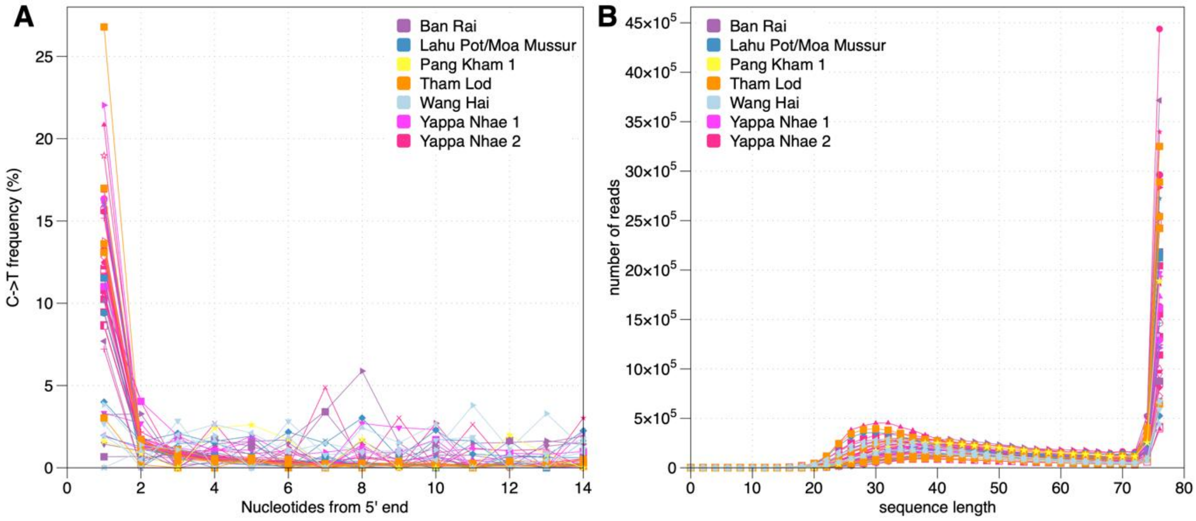

Supplementary Figure 2. Quality control plots of ancient genomes from seven archaeological sites in northwestern Thailand; **A**, frequency of C-T transitions at the 5' end of the DNA fragments; **B**, read length distribution; plotted with DataGraph v4.4 (Visual Data Tools, Inc., http://www.visualdatatools.com/DataGraph/). Source data are provided as a Source Data file.

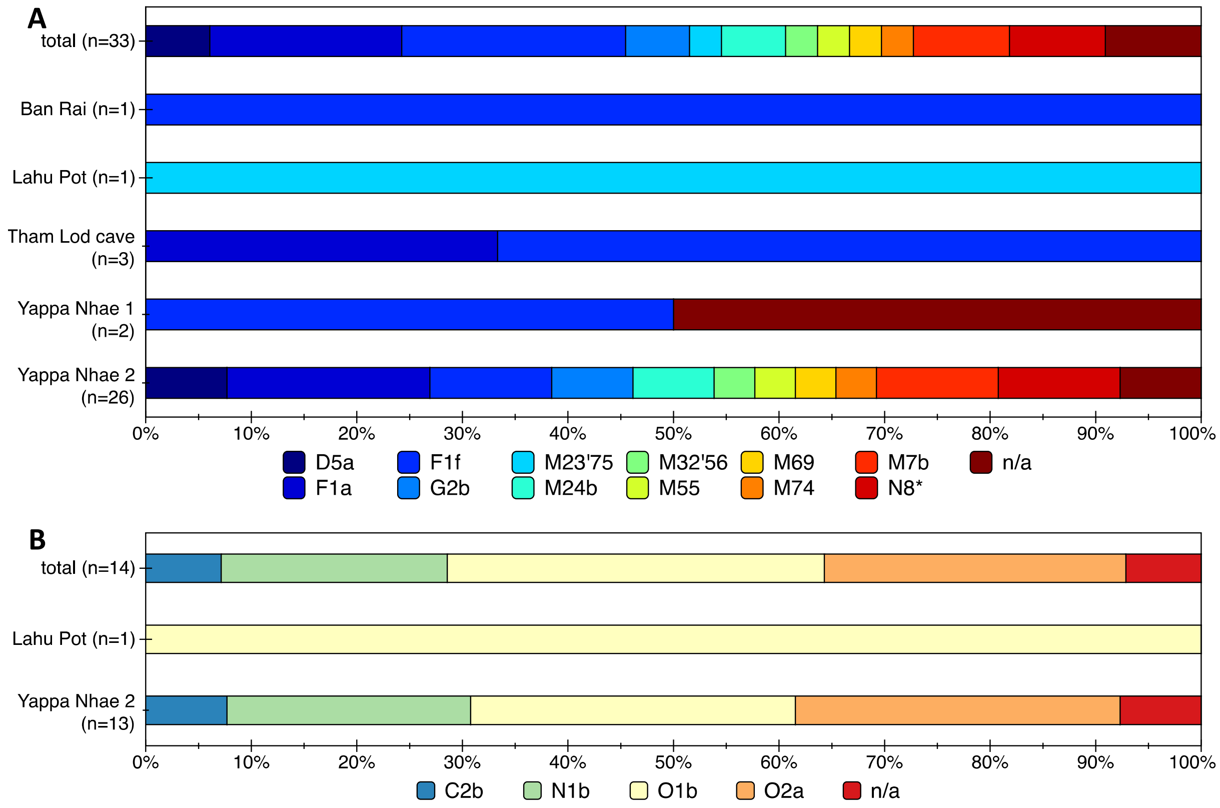

Supplementary Figure 3. The diversity of mitochondrial (**A**) and Y-haplogroups (**B**) of ancient Log Coffin-associated genomes from northwestern Thailand represented pooled across sites as well as for individual sites, plotted with DataGraph v4.4 (Visual Data Tools, Inc., http://www.visualdatatools.com/DataGraph/). Source data are provided as a Source Data file.

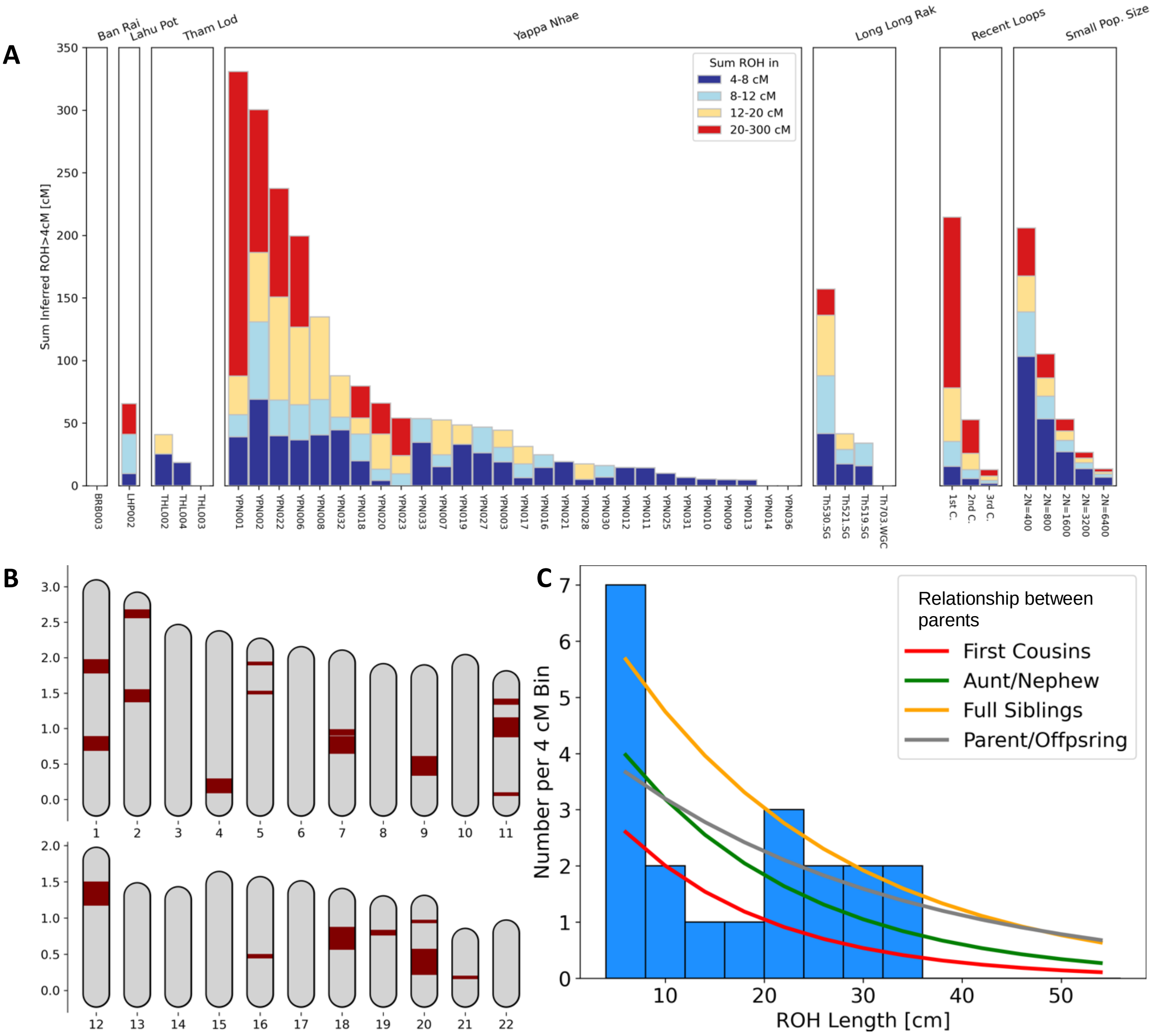

Supplementary Figure 4. Runs of homozygosity (ROH) of ancient individuals from northwestern Thailand: (**A**) lengths of ROH for each ancient individual, previously simulated data expected for di fferent parental relationships and population sizes displayed on the right[49], (**B**) location of ROH within the karyotype of YPN001 and (**C**) ROH length distribution of YPN001 (blue bars) with lines depicting the “expected” ROH trajectory of relationships between parents superimposed. Plotted with hapROH v0.6[53], source data are provided as a Source Data file.

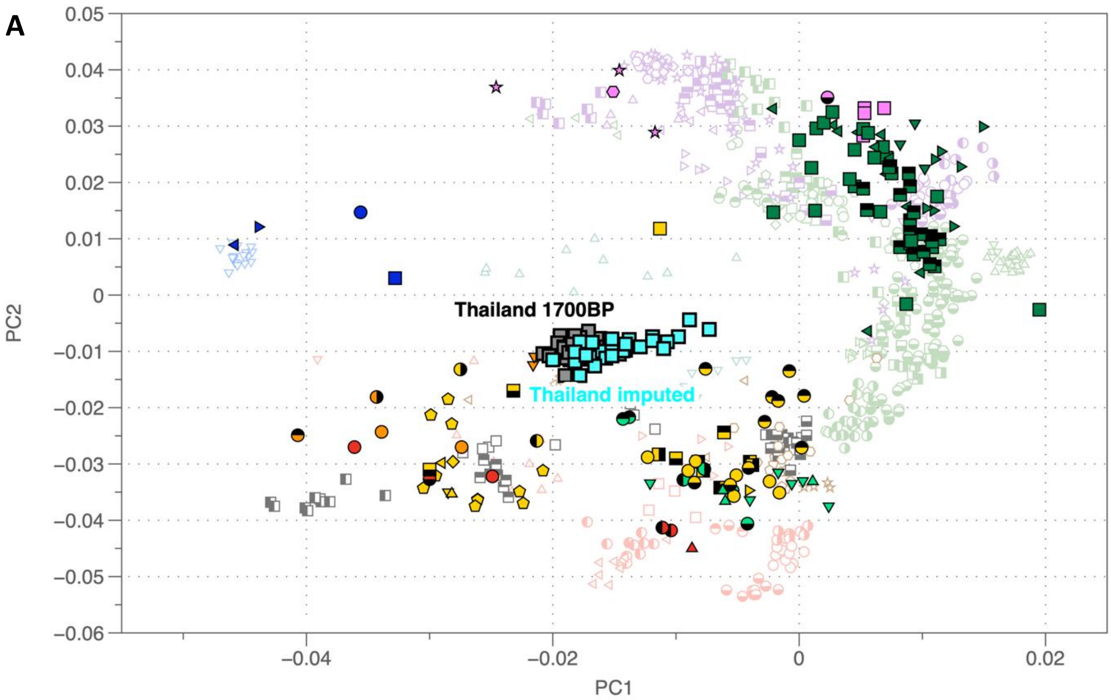

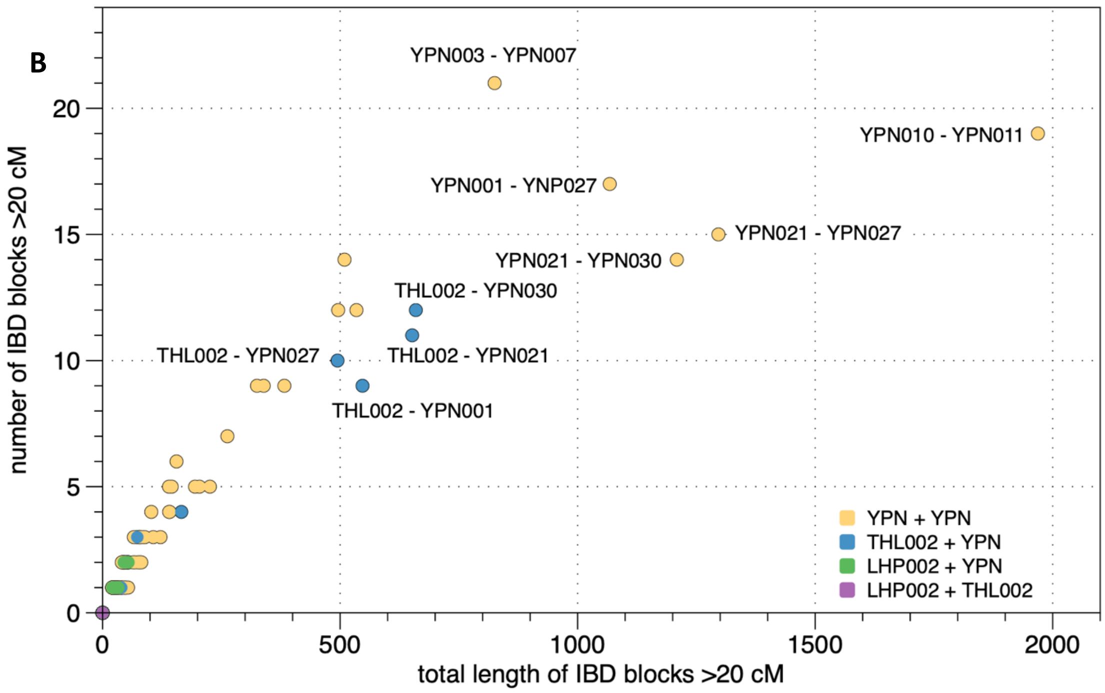

Supplementary Figure 5. (**A**) Principal component analyses calculated on present-day East and Southeast Asian individuals[45,46,48,49,55] showing the position of the Log Coffin-associated genomes before (grey squares) and after imputation (turquoise squares), full legend as in Supplementary Figure 6, source data are provided as a Source Data file; (**B**) Shared IBD blocks between newly generated Log Coffin-associated individuals from Thailand coloured by genetic relationships between individuals from different burial sites (Supplementary Data 4); plotted with DataGraph v4.4 (Visual Data Tools, Inc., http://www.visualdatatools.com/DataGraph/).

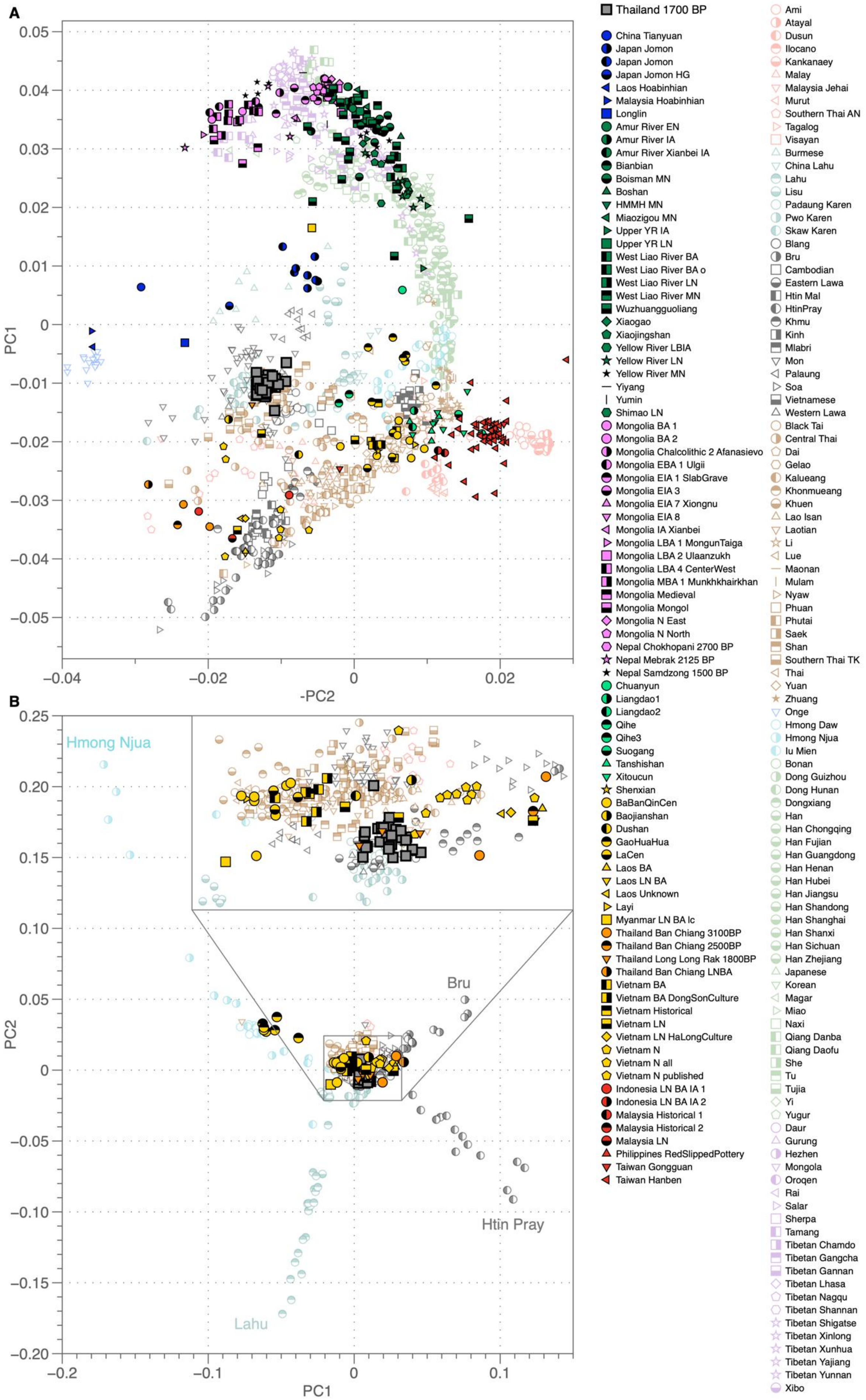

A
0.05
0.04
0.03
0.02
0.01
0
−0.01
−0.02
−0.03
−0.04
−0.05
PC1
−0.04
−0.02
0
0.02
-PC2
B
0.25
0.20
0.15
0.10
0.05
0
−0.05
−0.10
−0.15
−0.20
PC2
−0.2
−0.1
0
0.1
PC1
Hmong Njua
Bru
Htin Pray
Lahu
Thailand 1700 BP
China Tianyuan
Japan Jomon
Japan Jomon
Japan Jomon HG
Laos Hoabinhian
Malaysia Hoabinhian
Longlin
Amur River EN
Amur River IA
Amur River Xianbei IA
Bianbian
Boisman MN
Boshan
HMMH MN
Miaozigou MN
Upper YR IA
Upper YR LN
West Liao River BA
West Liao River BA o
West Liao River LN
West Liao River MN
Wuzhuangguoliang
Xiaogao
Xiaojingshan
Yellow River LBIA
Yellow River LN
Yellow River MN
Yiyang
Yumin
Shimao LN
Mongolia BA 1
Mongolia BA 2
Mongolia Chalcolithic 2 Afanasievo
Mongolia EBA 1 Ulgii
Mongolia EIA 1 SlabGrave
Mongolia EIA 3
Mongolia EIA 7 Xiongnu
Mongolia EIA 8
Mongolia IA Xianbei
Mongolia LBA 1 MongunTaiga
Mongolia LBA 2 Ulaanzukh
Mongolia LBA 4 CenterWest
Mongolia MBA 1 Munkhkhairkhan
Mongolia Medieval
Mongolia Mongol
Mongolia N East
Mongolia N North
Nepal Chokhopani 2700 BP
Nepal Mebrak 2125 BP
Nepal Samdzong 1500 BP
Chuanyun
Liangdao1
Liangdao2
Qihe
Qihe3
Suogang
Tanshishan
Xitoucun
Shenxian
BaBanQinCen
Baojianshan
Dushan
GaoHuaHua
LaCen
Laos BA
Laos LN BA
Laos Unknown
Layi
Myanmar LN BA Ic
Thailand Ban Chiang 3100BP
Thailand Ban Chiang 2500BP
Thailand Long Long Rak 1800BP
Thailand Ban Chiang LNBA
Vietnam BA
Vietnam BA DongSonCulture
Vietnam Historical
Vietnam LN
Vietnam LN HaLongCulture
Vietnam N
Vietnam N all
Vietnam N published
Indonesia LN BA IA 1
Indonesia LN BA IA 2
Malaysia Historical 1
Malaysia Historical 2
Malaysia LN
Philippines RedSlippedPottery
Taiwan Gongguan
Taiwan Hanben
Ami
Atayal
Dusun
Ilocano
Kankanaey
Malay
Malaysia Jehai
Murut
Southern Thai AN
Tagalog
Visayan
Burmese
China Lahu
Lahu
Lisu
Padaung Karen
Pwo Karen
Skaw Karen
Blang
Bru
Cambodian
Eastern Lawa
Htin Mal
HtinPray
Khmu
Kinh
Mlabri
Mon
Palaung
Soa
Vietnamese
Western Lawa
Black Tai
Central Thai
Dai
Gelao
Kalueang
Khonmueang
Khuen
Lao Isan
Laotian
Li
Lue
Maonan
Mulam
Nyaw
Phuan
Phutai
Saek
Shan
Southern Thai TK
Thai
Yuan
Zhuang
Onge
Hmong Daw
Hmong Njua
Iu Mien
Bonan
Dong Guizhou
Dong Hunan
Dongxiang
Han
Han Chongqing
Han Fujian
Han Guangdong
Han Henan
Han Hubei
Han Jiangsu
Han Shandong
Han Shanghai
Han Shanxi
Han Sichuan
Han Zhejiang
Japanese
Korean
Magar
Miao
Naxi
Qiang Danba
Qiang Daofu
She
Tu
Tujia
Yi
Yugur
Daur
Gurung
Hezhen
Mongola
Oroqen
Rai
Salar
Sherpa
Tamang
Tibetan Chamdo
Tibetan Gangcha
Tibetan Gannan
Tibetan Lhasa
Tibetan Nagqu
Tibetan Shannan
Tibetan Shigatse
Tibetan Xinlong
Tibetan Xunhua
Tibetan Yajiang
Tibetan Yunnan
Xibo

Supplementary Figure 6. Principal component analyses calculated on (**A**) present-day East Asian individuals[45,46,48,49,53,55] and (**B**) present-day individuals from Thailand[49]; plotted with DataGraph v4.4 (Visual Data Tools, Inc., http://www.visualdatatools.com/DataGraph/). Source data are provided as a Source Data file.

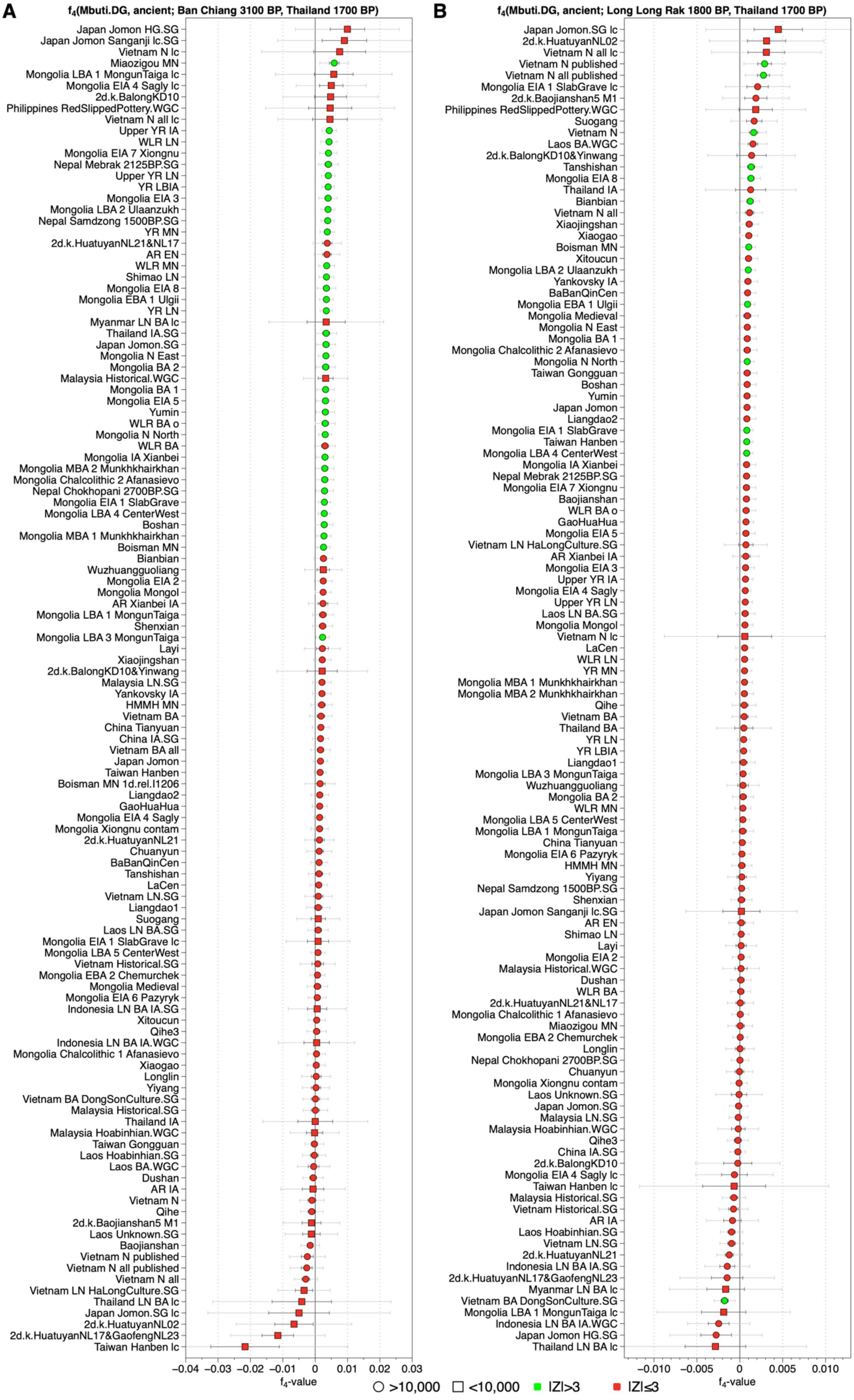
A
f4(Mbuti.DG, ancient; Ban Chiang 3100 BP, Thailand 1700 BP)
Japan Jomon HG.SG
Japan Jomon Sanganji Ic.SG
Vietnam N Ic
Miaozigou MN
Mongolia LBA 1 MongunTaiga Ic
Mongolia EIA 4 Sagly Ic
2d.k.BalongKD10
Philippines RedSlippedPottery.WGC
Vietnam N all Ic
Upper YR IA
WLR LN
Mongolia EIA 7 Xiongnu
Nepal Mebrak 2125BP.SG
Upper YR LN
YR LBIA
Mongolia EIA 3
Mongolia LBA 2 Ulaanzukh
Nepal Samdzong 1500BP.SG
YR MN
2d.k.HuatuyanNL21&NL17
AR EN
WLR MN
Shimao LN
Mongolia EIA 8
Mongolia EBA 1 Ulgii
YR LN
Myanmar LN BA Ic
Thailand IA.SG
Japan Jomon.SG
Mongolia N East
Mongolia BA 2
Malaysia Historical.WGC
Mongolia BA 1
Mongolia EIA 5
Yumin
WLR BA o
Mongolia N North
WLR BA
Mongolia IA Xianbei
Mongolia MBA 2 Munkhkhairkhan
Mongolia Chalcolithic 2 Afanasievo
Nepal Chokhopani 2700BP.SG
Mongolia EIA 1 SlabGrave
Mongolia LBA 4 CenterWest
Boshan
Mongolia MBA 1 Munkhkhairkhan
Boisman MN
Bianbian
Wuzhuangguoliang
Mongolia EIA 2
Mongolia Mongol
AR Xianbei IA
Mongolia LBA 1 MongunTaiga
Shenxian
Mongolia LBA 3 MongunTaiga
Layi
Xiaojingshan
2d.k.BalongKD10&Yinwang
Malaysia LN.SG
Yankovsky IA
HMMH MN
Vietnam BA
China Tianyuan
China IA.SG
Vietnam BA all
Japan Jomon
Taiwan Hanben
Boisman MN 1d.rel.I1206
Liangdao2
GaoHuaHua
Mongolia EIA 4 Sagly
Mongolia Xiongnu contam
2d.k.HuatuyanNL21
Chuanyun
BaBanQinCen
Tanshishan
LaCen
Vietnam LN.SG
Liangdao1
Suogang
Laos LN BA.SG
Mongolia EIA 1 SlabGrave Ic
Mongolia LBA 5 CenterWest
Vietnam Historical.SG
Mongolia EBA 2 Chemurchek
Mongolia Medieval
Mongolia EIA 6 Pazyryk
Indonesia LN BA IA.SG
Xitoucun
Qihe3
Indonesia LN BA IA.WGC
Mongolia Chalcolithic 1 Afanasievo
Xiaogao
Longlin
Yiyang
Vietnam BA DongSonCulture.SG
Malaysia Historical.SG
Thailand IA
Malaysia Hoabinhian.WGC
Taiwan Gongguan
Laos Hoabinhian.SG
Laos BA.WGC
Dushan
AR IA
Vietnam N
Qihe
2d.k.Baojianshan5 M1
Laos Unknown.SG
Baojianshan
Vietnam N published
Vietnam N all published
Vietnam N all
Vietnam LN HaLongCulture.SG
Thailand LN BA Ic
Japan Jomon.SG Ic
2d.k.HuatuyanNL02
2d.k.HuatuyanNL17&GaofengNL23
Taiwan Hanben Ic
−0.04 −0.03 −0.02 −0.01 0 0.01 0.02 0.03
f4-value
B
f4(Mbuti.DG, ancient; Long Long Rak 1800 BP, Thailand 1700 BP)
Japan Jomon.SG Ic
2d.k.HuatuyanNL02
Vietnam N all Ic
Vietnam N published
Vietnam N all published
Mongolia EIA 1 SlabGrave Ic
2d.k.Baojianshan5 M1
Philippines RedSlippedPottery.WGC
Suogang
Vietnam N
Laos BA.WGC
2d.k.BalongKD10&Yinwang
Tanshishan
Mongolia EIA 8
Thailand IA
Bianbian
Vietnam N all
Xiaojingshan
Xiaogao
Boisman MN
Xitoucun
Mongolia LBA 2 Ulaanzukh
Yankovsky IA
BaBanQinCen
Mongolia EBA 1 Ulgii
Mongolia Medieval
Mongolia N East
Mongolia BA 1
Mongolia Chalcolithic 2 Afanasievo
Mongolia N North
Taiwan Gongguan
Boshan
Yumin
Japan Jomon
Liangdao2
Mongolia EIA 1 SlabGrave
Taiwan Hanben
Mongolia LBA 4 CenterWest
Mongolia IA Xianbei
Nepal Mebrak 2125BP.SG
Mongolia EIA 7 Xiongnu
Baojianshan
WLR BA o
GaoHuaHua
Mongolia EIA 5
Vietnam LN HaLongCulture.SG
AR Xianbei IA
Mongolia EIA 3
Upper YR IA
Mongolia EIA 4 Sagly
Upper YR LN
Laos LN BA.SG
Mongolia Mongol
Vietnam N Ic
LaCen
WLR LN
YR MN
Mongolia MBA 1 Munkhkhairkhan
Mongolia MBA 2 Munkhkhairkhan
Qihe
Vietnam BA
Thailand BA
YR LN
YR LBIA
Liangdao1
Mongolia LBA 3 MongunTaiga
Wuzhuangguoliang
Mongolia BA 2
WLR MN
Mongolia LBA 5 CenterWest
Mongolia LBA 1 MongunTaiga
China Tianyuan
Mongolia EIA 6 Pazyryk
HMMH MN
Yiyang
Nepal Samdzong 1500BP.SG
Shenxian
Japan Jomon Sanganji Ic.SG
AR EN
Shimao LN
Layi
Mongolia EIA 2
Malaysia Historical.WGC
Dushan
WLR BA
2d.k.HuatuyanNL21&NL17
Mongolia Chalcolithic 1 Afanasievo
Miaozigou MN
Mongolia EBA 2 Chemurchek
Longlin
Nepal Chokhopani 2700BP.SG
Chuanyun
Mongolia Xiongnu contam
Laos Unknown.SG
Japan Jomon.SG
Malaysia LN.SG
Malaysia Hoabinhian.WGC
Qihe3
China IA.SG
2d.k.BalongKD10
Mongolia EIA 4 Sagly Ic
Taiwan Hanben Ic
Malaysia Historical.SG
Vietnam Historical.SG
AR IA
Laos Hoabinhian.SG
Vietnam LN.SG
2d.k.HuatuyanNL21
Indonesia LN BA IA.SG
2d.k.HuatuyanNL17&GaofengNL23
Myanmar LN BA Ic
Vietnam BA DongSonCulture.SG
Mongolia LBA 1 MongunTaiga Ic
Indonesia LN BA IA.WGC
Japan Jomon HG.SG
Thailand LN BA Ic
−0.010 −0.005 0 0.005 0.010
f4-value
○ >10,000 □ <10,000 ■ |Z|>3 ■ |Z|≤3

Supplementary Figure 7. Observed f_4-statistics comparing the genetic affinities of the newly genotyped individuals from northwestern Thailand with (**A**) Bronze Age northeastern Thailand[50] and (**B**) Iron Age northwestern Thailand[44]; red and green indicate z-scores such that |Z|≤3 and |Z|>3 respectively, circles and squares denote f_4-statistics where there were more and less than 10,000 overlapping SNPs respectively, dark grey lines represent ± 1 standard error as calculated by the qpDstat[45], light grey lines represent ± 3 standard errors; plotted with DataGraph v4.4 (Visual Data Tools, Inc., http://www.visualdatatools.com/DataGraph/). Source data are provided as a Source Data file.

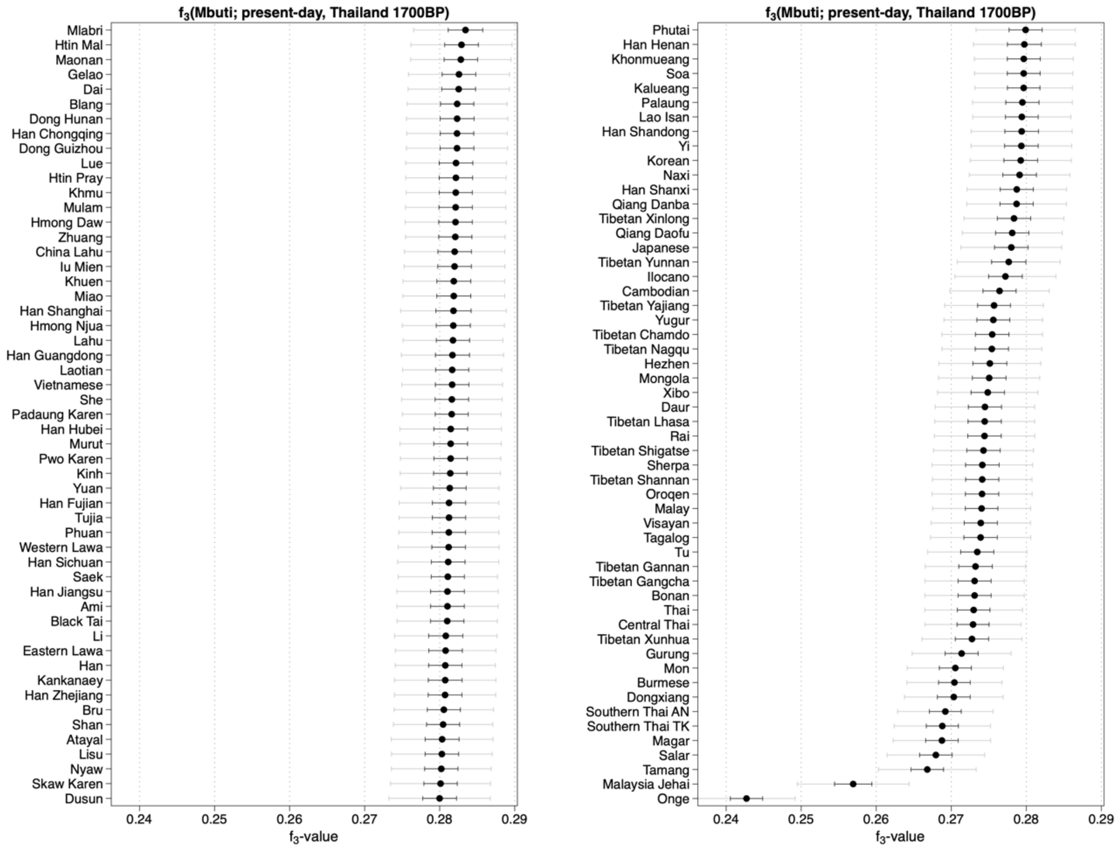

Supplementary Figure 8. Observed f_3-stastics showing shared genetic drift between ancient genomes from northwestern Thailand and present-day groups from the region[44–46,49,53,55], dark grey lines represent ± 1 standard error as calculated by the qp3Pop[45], light grey lines represent ± 3 standard errors, plotted with DataGraph v4.4 (Visual Data Tools, Inc., http://www.visualdatatools.com/DataGraph/). Source data are provided as a Source Data file.

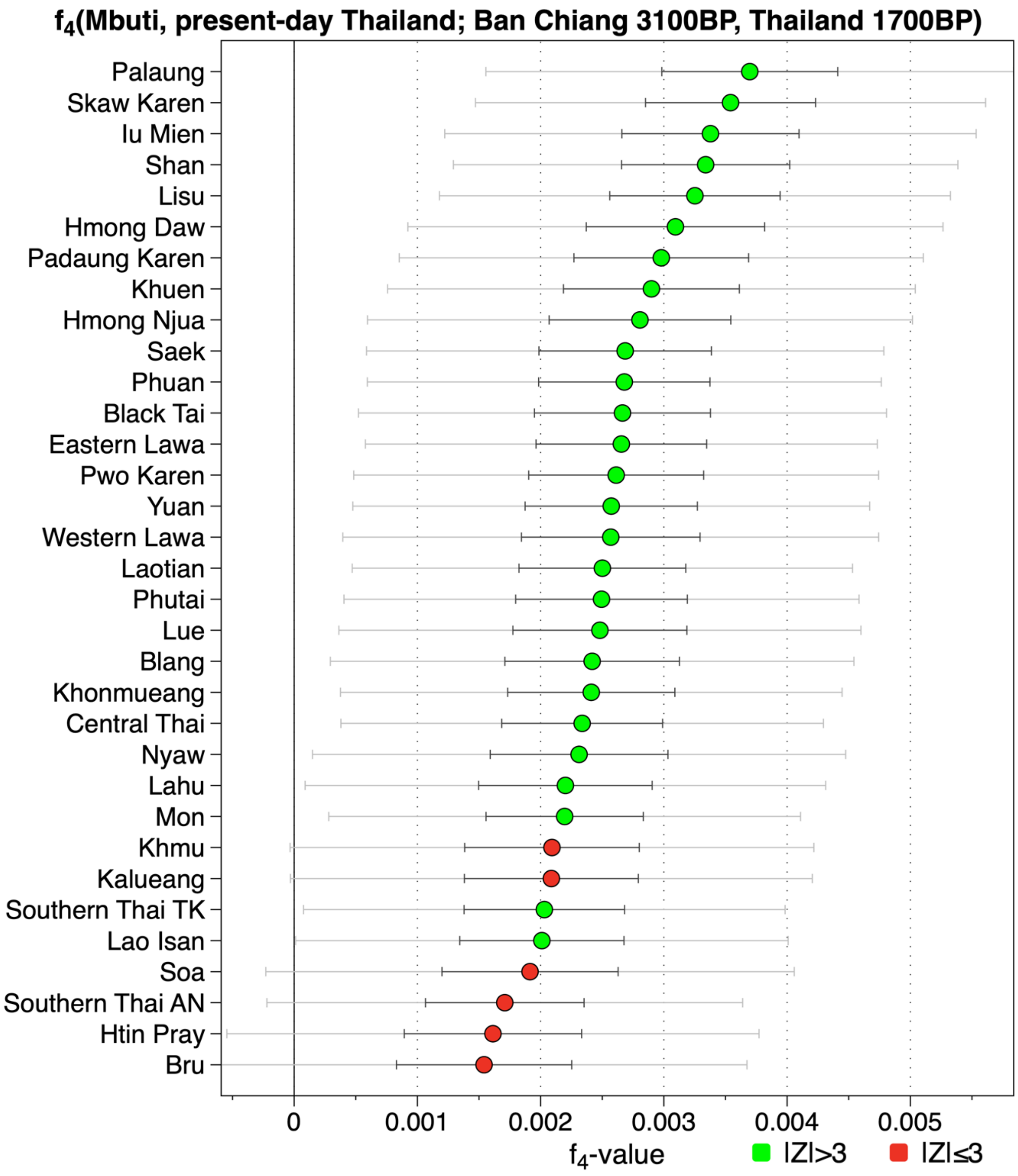

Supplementary Figure 9. Observed f_4-statistics comparing the affinity of the newly generated genomes from ancient northwestern Thailand and Bronze Age northeastern Thailand [52] to present-day groups from Thailand[50], red and green indicate z-scores such that $|Z| \leq 3$ and $|Z|>3$ respectively, dark grey lines represent ± 1 standard error as calculated by the qpDstat[45], light grey lines represent ± 3 standard errors, plotted with DataGraph v4.4 (Visual Data Tools, Inc., http://www.visualdatatools.com/DataGraph/). Source data are provided as a Source Data file.

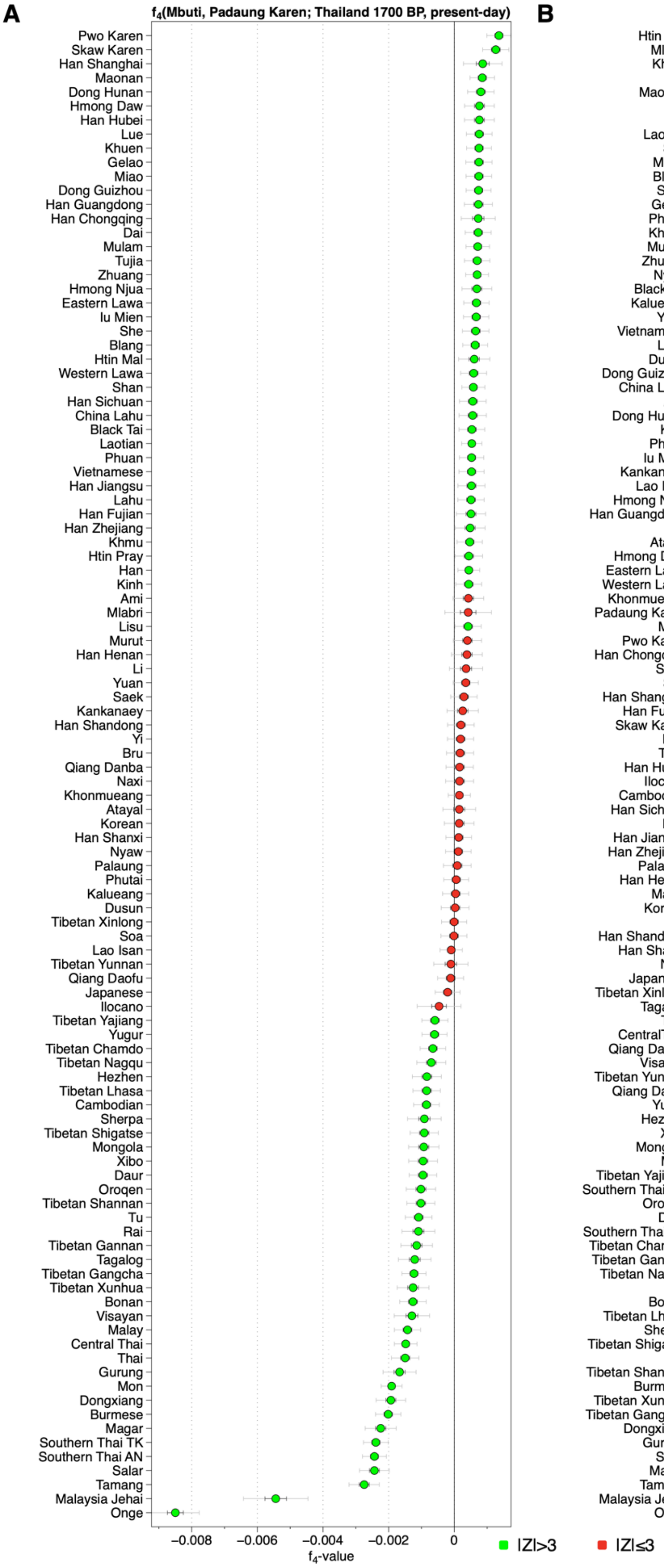
A
f_4(Mbuti, Padaung Karen; Thailand 1700 BP, present-day)
Pwo Karen
Skaw Karen
Han Shanghai
Maonan
Dong Hunan
Hmong Daw
Han Hubei
Lue
Khuen
Gelao
Miao
Dong Guizhou
Han Guangdong
Han Chongqing
Dai
Mulam
Tujia
Zhuang
Hmong Njua
Eastern Lawa
Iu Mien
She
Blang
Htin Mal
Western Lawa
Shan
Han Sichuan
China Lahu
Black Tai
Laotian
Phuan
Vietnamese
Han Jiangsu
Lahu
Han Fujian
Han Zhejiang
Khmu
Htin Pray
Han
Kinh
Ami
Mlabri
Lisu
Murut
Han Henan
Li
Yuan
Saek
Kankanaey
Han Shandong
Yi
Bru
Qiang Danba
Naxi
Khonmueang
Atayal
Korean
Han Shanxi
Nyaw
Palaung
Phutai
Kalueang
Dusun
Tibetan Xinlong
Soa
Lao Isan
Tibetan Yunnan
Qiang Daofu
Japanese
Ilocano
Tibetan Yajiang
Yugur
Tibetan Chamdo
Tibetan Nagqu
Hezhen
Tibetan Lhasa
Cambodian
Sherpa
Tibetan Shigatse
Mongola
Xibo
Daur
Oroqen
Tibetan Shannan
Tu
Rai
Tibetan Gannan
Tagalog
Tibetan Gangcha
Tibetan Xunhua
Bonan
Visayan
Malay
Central Thai
Thai
Gurung
Mon
Dongxiang
Burmese
Magar
Southern Thai TK
Southern Thai AN
Salar
Tamang
Malaysia Jehai
Onge
−0.008
−0.006
−0.004
−0.002
0
f_4-value
B
f_4(Mbuti, Htin Pray; Thailand 1700 BP, present-day)
Htin Mal
Mlabri
Khmu
Bru
Maonan
Dai
Lue
Laotian
Soa
Murut
Blang
Saek
Gelao
Phuan
Khuen
Mulam
Zhuang
Nyaw
Black Tai
Kalueang
Yuan
Vietnamese
Lahu
Dusun
Dong Guizhou
China Lahu
Ami
Dong Hunan
Kinh
Phutai
Iu Mien
Kankanaey
Lao Isan
Hmong Njua
Han Guangdong
Li
Atayal
Hmong Daw
Eastern Lawa
Western Lawa
Khonmueang
Padaung Karen
Miao
Pwo Karen
Han Chongqing
Shan
She
Han Shanghai
Han Fujian
Skaw Karen
Lisu
Tujia
Han Hubei
Ilocano
Cambodian
Han Sichuan
Han
Han Jiangsu
Han Zhejiang
Palaung
Han Henan
Malay
Korean
Yi
Han Shandong
Han Shanxi
Naxi
Japanese
Tibetan Xinlong
Tagalog
Thai
CentralThai
Qiang Danba
Visayan
Tibetan Yunnan
Qiang Daofu
Yugur
Hezhen
Xibo
Mongola
Mon
Tibetan Yajiang
Southern Thai AN
Oroqen
Daur
Southern Thai TK
Tibetan Chamdo
Tibetan Gannan
Tibetan Nagqu
Tu
Bonan
Tibetan Lhasa
Sherpa
Tibetan Shigatse
Rai
Tibetan Shannan
Burmese
Tibetan Xunhua
Tibetan Gangcha
Dongxiang
Gurung
Salar
Magar
Tamang
Malaysia Jehai
Onge
−0.008
−0.006
−0.004
−0.002
0
0.002
f_4-value
|Z|>3
|Z|≤3

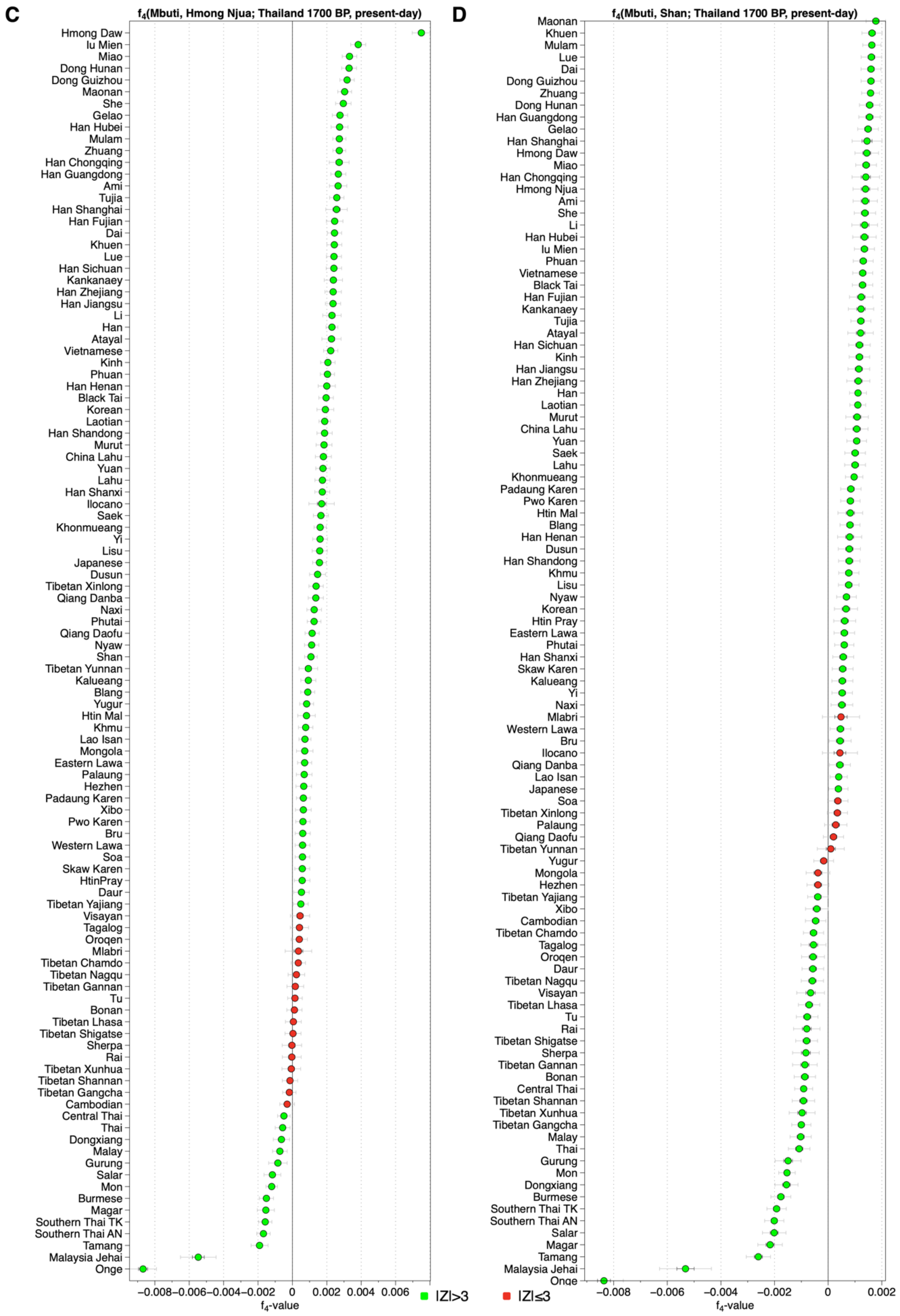
C
f4(Mbuti, Hmong Njua; Thailand 1700 BP, present-day)
Hmong Daw
Iu Mien
Miao
Dong Hunan
Dong Guizhou
Maonan
She
Gelao
Han Hubei
Mulam
Zhuang
Han Chongqing
Han Guangdong
Ami
Tujia
Han Shanghai
Han Fujian
Dai
Khuen
Lue
Han Sichuan
Kankanaey
Han Zhejiang
Han Jiangsu
Li
Han
Atayal
Vietnamese
Kinh
Phuan
Han Henan
Black Tai
Korean
Laotian
Han Shandong
Murut
China Lahu
Yuan
Lahu
Han Shanxi
Ilocano
Saek
Khonmueang
Yi
Lisu
Japanese
Dusun
Tibetan Xinlong
Qiang Danba
Naxi
Phutai
Qiang Daofu
Nyaw
Shan
Tibetan Yunnan
Kalueang
Blang
Yugur
Htin Mal
Khmu
Lao Isan
Mongola
Eastern Lawa
Palaung
Hezhen
Padaung Karen
Xibo
Pwo Karen
Bru
Western Lawa
Soa
Skaw Karen
HtinPray
Daur
Tibetan Yajiang
Visayan
Tagalog
Oroqen
Mlabri
Tibetan Chamdo
Tibetan Nagqu
Tibetan Gannan
Tu
Bonan
Tibetan Lhasa
Tibetan Shigatse
Sherpa
Rai
Tibetan Xunhua
Tibetan Shannan
Tibetan Gangcha
Cambodian
Central Thai
Thai
Dongxiang
Malay
Gurung
Salar
Mon
Burmese
Magar
Southern Thai TK
Southern Thai AN
Tamang
Malaysia Jehai
Onge
−0.008 −0.006 −0.004 −0.002 0 0.002 0.004 0.006
f4-value
D
f4(Mbuti, Shan; Thailand 1700 BP, present-day)
Maonan
Khuen
Mulam
Lue
Dai
Dong Guizhou
Zhuang
Dong Hunan
Han Guangdong
Gelao
Han Shanghai
Hmong Daw
Miao
Han Chongqing
Hmong Njua
Ami
She
Li
Han Hubei
Iu Mien
Phuan
Vietnamese
Black Tai
Han Fujian
Kankanaey
Tujia
Atayal
Han Sichuan
Kinh
Han Jiangsu
Han Zhejiang
Han
Laotian
Murut
China Lahu
Yuan
Saek
Lahu
Khonmueang
Padaung Karen
Pwo Karen
Htin Mal
Blang
Han Henan
Dusun
Han Shandong
Khmu
Lisu
Nyaw
Korean
Htin Pray
Eastern Lawa
Phutai
Han Shanxi
Skaw Karen
Kalueang
Yi
Naxi
Mlabri
Western Lawa
Bru
Ilocano
Qiang Danba
Lao Isan
Japanese
Soa
Tibetan Xinlong
Palaung
Qiang Daofu
Tibetan Yunnan
Yugur
Mongola
Hezhen
Tibetan Yajiang
Xibo
Cambodian
Tibetan Chamdo
Tagalog
Oroqen
Daur
Tibetan Nagqu
Visayan
Tibetan Lhasa
Tu
Rai
Tibetan Shigatse
Sherpa
Tibetan Gannan
Bonan
Central Thai
Tibetan Shannan
Tibetan Xunhua
Tibetan Gangcha
Malay
Thai
Gurung
Mon
Dongxiang
Burmese
Southern Thai TK
Southern Thai AN
Salar
Magar
Tamang
Malaysia Jehai
Onge
−0.008 −0.006 −0.004 −0.002 0 0.002
f4-value
|Z|>3
|Z|≤3

Supplementary Figure 10. Observed f_4-statistics comparing the affinity of the newly generated genomes from ancient northwestern Thailand to present-day groups[44–46,49,53,55] and hill tribes from Thailand[53] speaking Sino-Tibetan (**A**), Austroasiatic (**B**), Hmong Mien (**C**) and Tai-Kadai (**D**) languages, red and green indicate z-scores such that $|Z|\leq 3$ and $|Z|>3$ respectively, dark grey lines represent ± 1 standard error as calculated by the qpDstat[45], light grey lines represent ± 3 standard errors, plotted with DataGraph v4.4 (Visual Data Tools, Inc., http://www.visualdatatools.com/DataGraph/). Source data are provided as a Source Data file.

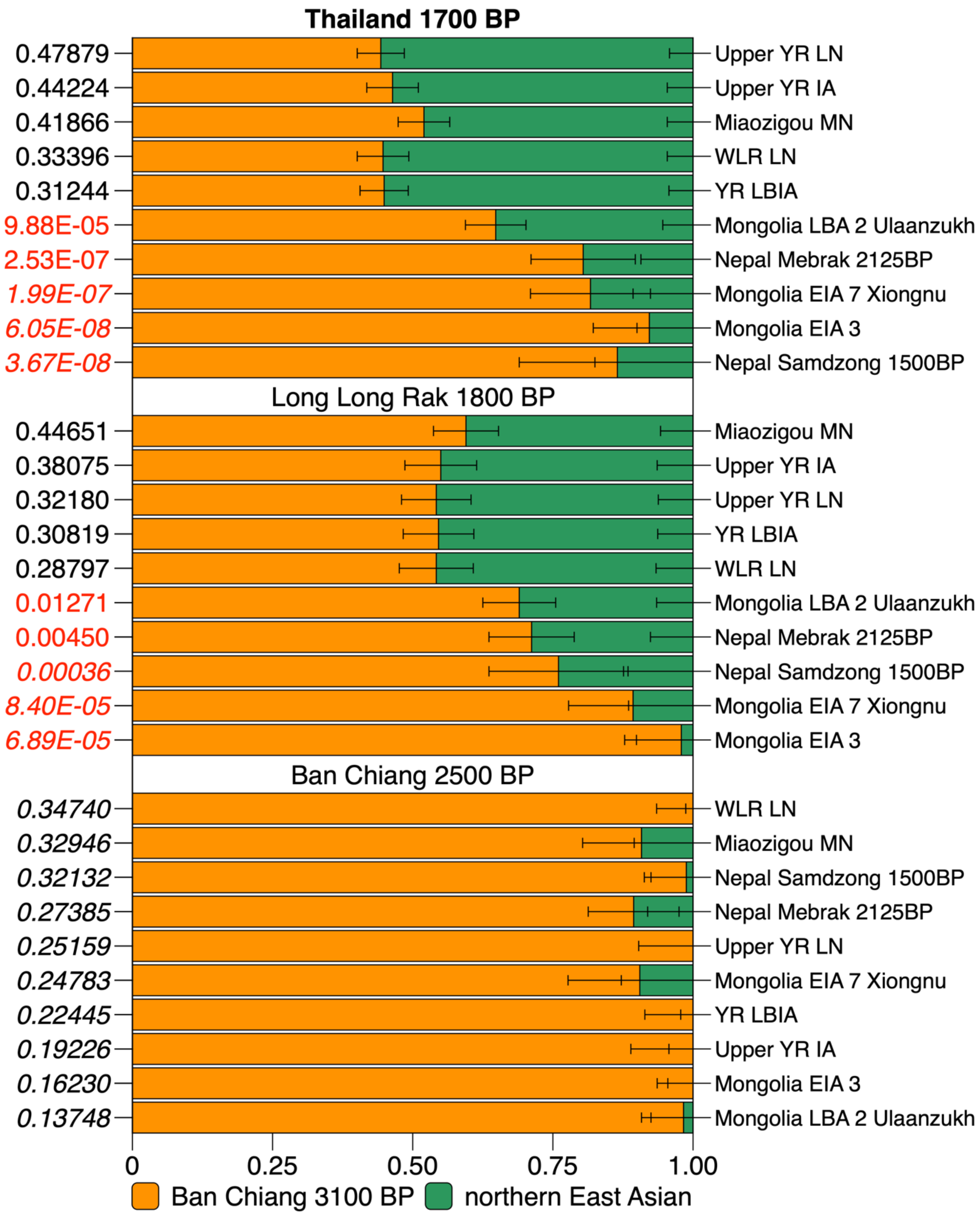

Thailand 1700 BP
0.47879 Upper YR LN
0.44224 Upper YR IA
0.41866 Miaozigou MN
0.33396 WLR LN
0.31244 YR LBIA
9.88E-05 Mongolia LBA 2 Ulaanzukh
2.53E-07 Nepal Mebrak 2125BP
1.99E-07 Mongolia EIA 7 Xiongnu
6.05E-08 Mongolia EIA 3
3.67E-08 Nepal Samdzong 1500BP
Long Long Rak 1800 BP
0.44651 Miaozigou MN
0.38075 Upper YR IA
0.32180 Upper YR LN
0.30819 YR LBIA
0.28797 WLR LN
0.01271 Mongolia LBA 2 Ulaanzukh
0.00450 Nepal Mebrak 2125BP
0.00036 Nepal Samdzong 1500BP
8.40E-05 Mongolia EIA 7 Xiongnu
6.89E-05 Mongolia EIA 3
Ban Chiang 2500 BP
0.34740 WLR LN
0.32946 Miaozigou MN
0.32132 Nepal Samdzong 1500BP
0.27385 Nepal Mebrak 2125BP
0.25159 Upper YR LN
0.24783 Mongolia EIA 7 Xiongnu
0.22445 YR LBIA
0.19226 Upper YR IA
0.16230 Mongolia EIA 3
0.13748 Mongolia LBA 2 Ulaanzukh
0
0.25
0.50
0.75
1.00
Ban Chiang 3100 BP
northern East Asian

Supplementary Figure 11. Results of qpAdm analyses modelling ancient individuals from Iron Age northeastern and -western Thailand[44,49] as an admixture between Bronze Age northeastern Thailand[62] and ancient groups from northern East Asia[50]; p-values are given on the left where red indicates $p<0.05$ and italicized values indicate nested $p>0.05$, northern East Asian groups are displayed on the left, error bars in grey show ± 1 standard error as calculated with block jackknife in qpAdm v.1520[45]; plotted with DataGraph v4.4 (Visual Data Tools, Inc., http://www.visualdatatools.com/DataGraph/). Source data are provided as a Source Data file.

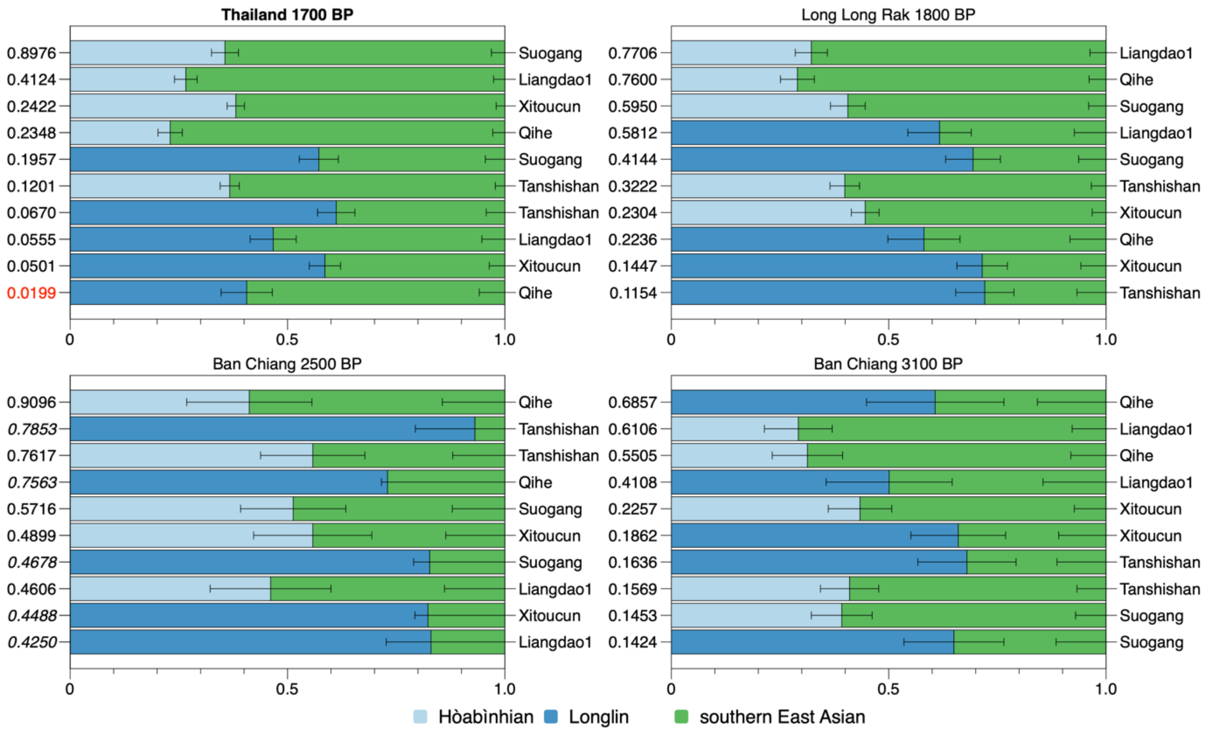

Supplementary Figure 12. Results of qpAdm analyses modelling the ancestry of ancient genomes from Thailand as an admixture between the Hòabìnhian-associated individual from Laos[44], Longlin[54] and southern East Asian individuals[52]; p-values are displayed on the left, where red indicates p<0.05 and italicized values indicate nested p>0.05, the southern East Asian sources are displayed on the right, error bars in grey show ± 1 standard error as calculated with block jackknife in qpAdm v.1520[45]; plotted with DataGraph v4.4 (Visual Data Tools, Inc., http://www.visualdatatools.com/DataGraph/). Source data are provided as a Source Data file.

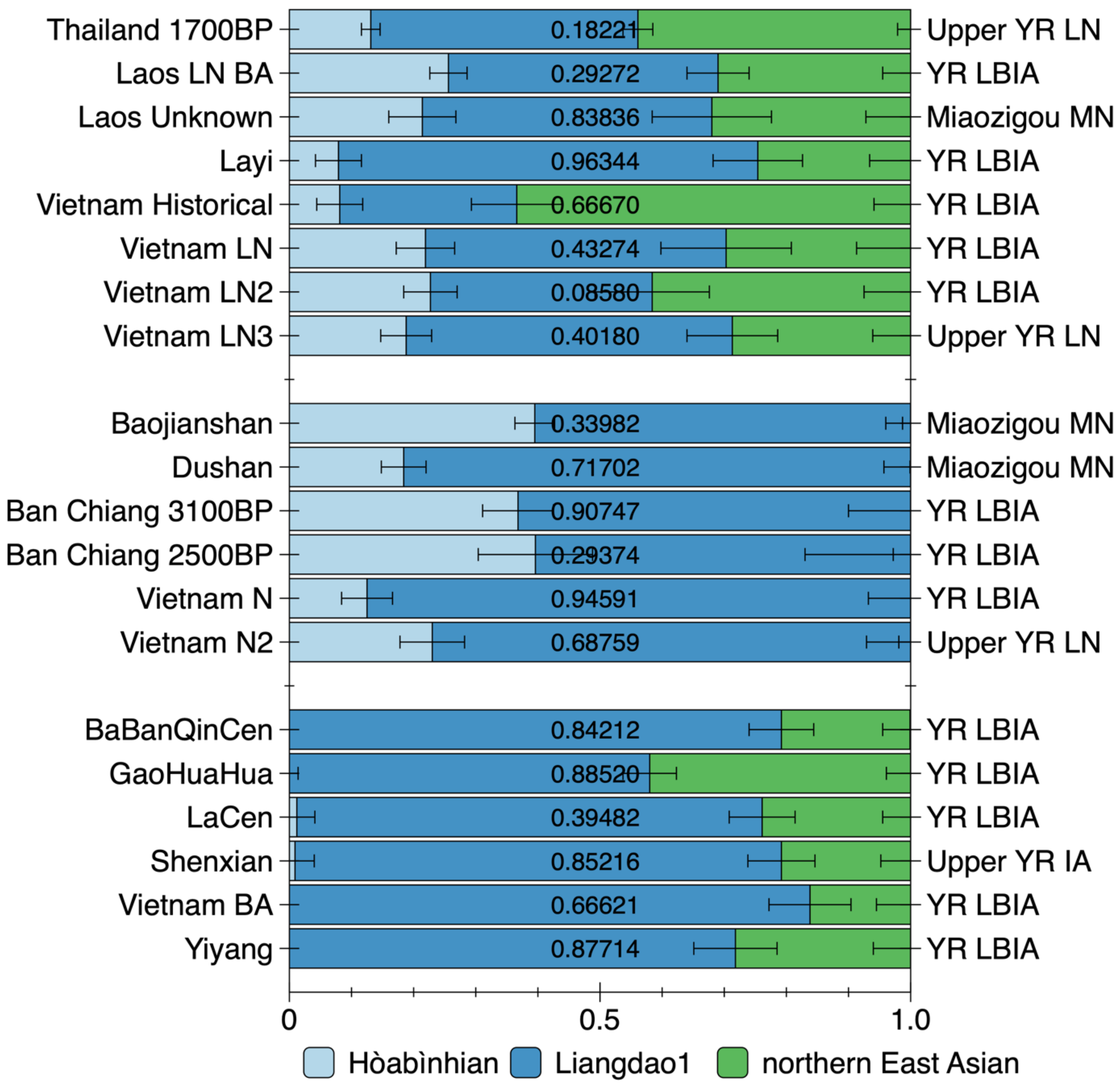

Supplementary Figure 13. Results of qpAdm analyses modelling ancient individuals from Southeast Asia as a three-way admixture between hunter-gatherers (light blue), ancient southern (light green), and ancient northern East Asian groups (dark green); p-values are displayed within the bars, northern East Asian sources on the left, error bars in grey show ± 1 standard error as calculated with block jackknife in qpAdm v.1520[45]; plotted with DataGraph v4.4 (Visual Data Tools, Inc., http://www.visualdatatools.com/DataGraph/). Source data are provided as a Source Data file.

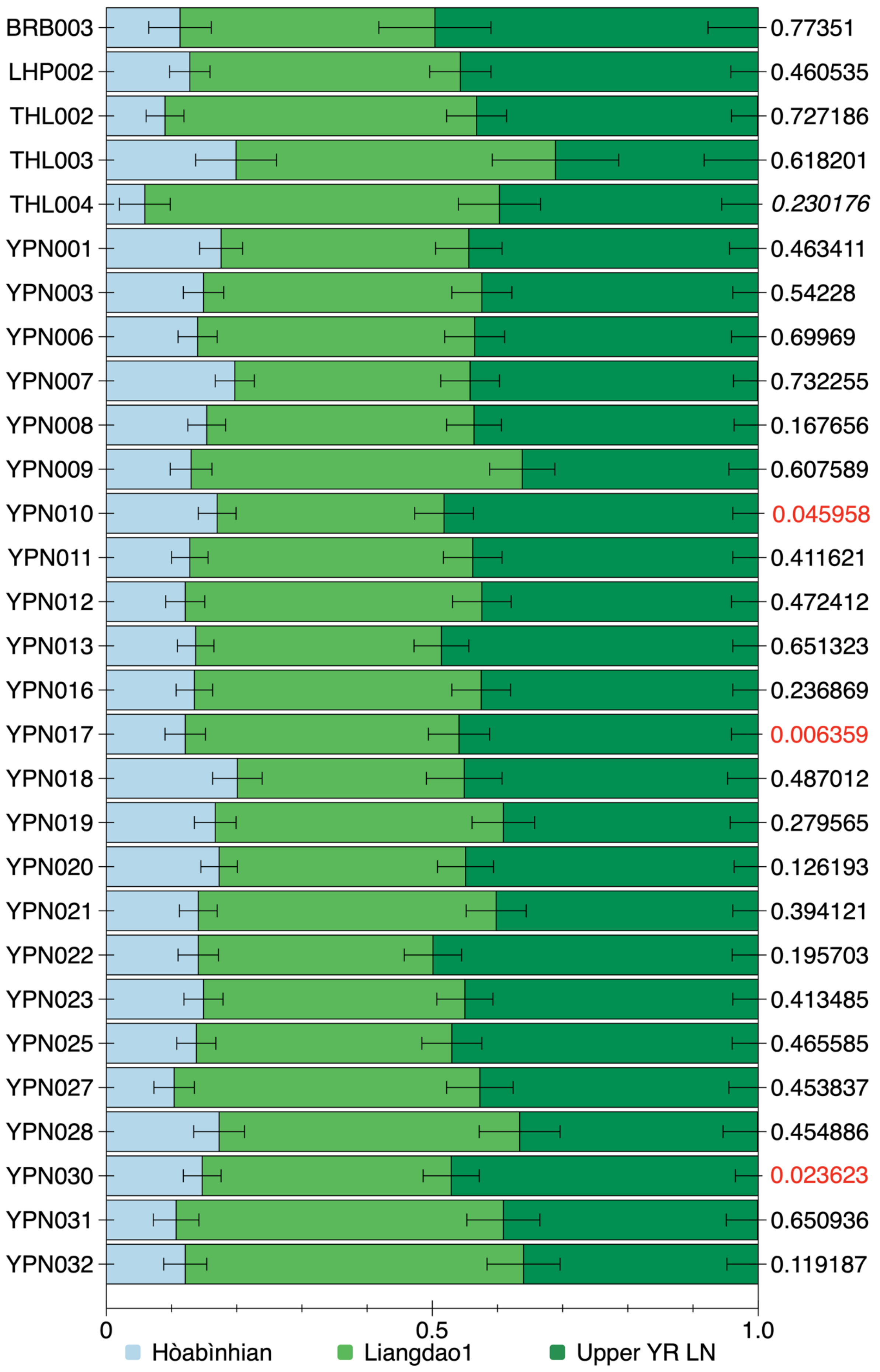
BRB003
LHP002
THL002
THL003
THL004
YPN001
YPN003
YPN006
YPN007
YPN008
YPN009
YPN010
YPN011
YPN012
YPN013
YPN016
YPN017
YPN018
YPN019
YPN020
YPN021
YPN022
YPN023
YPN025
YPN027
YPN028
YPN030
YPN031
YPN032
0.77351
0.460535
0.727186
0.618201
0.230176
0.463411
0.54228
0.69969
0.732255
0.167656
0.607589
0.045958
0.411621
0.472412
0.651323
0.236869
0.006359
0.487012
0.279565
0.126193
0.394121
0.195703
0.413485
0.465585
0.453837
0.454886
0.023623
0.650936
0.119187
0
0.5
1.0
Hòabìnhian
Liangdao1
Upper YR LN

Supplementary Figure 14. Results of qpAdm analyses modelling the newly generated ancient genomes from northwestern Thailand as an admixture of Hòabìnhian-associated individual from Laos[44], Liangdao1[52] and Upper Yellow River Late Neolithic (Upper YR LN)[50]; p-values are displayed on the right, where red numbers indicate $p<0.05$ and italic nested $p>0.05$, error bars in grey show ± 1 standard error as calculated with block jackknife in qpAdm v.1520[45]; plotted with DataGraph v4.4 (Visual Data Tools, Inc., http://www.visualdatatools.com/DataGraph/). Source data are provided as a Source Data file.

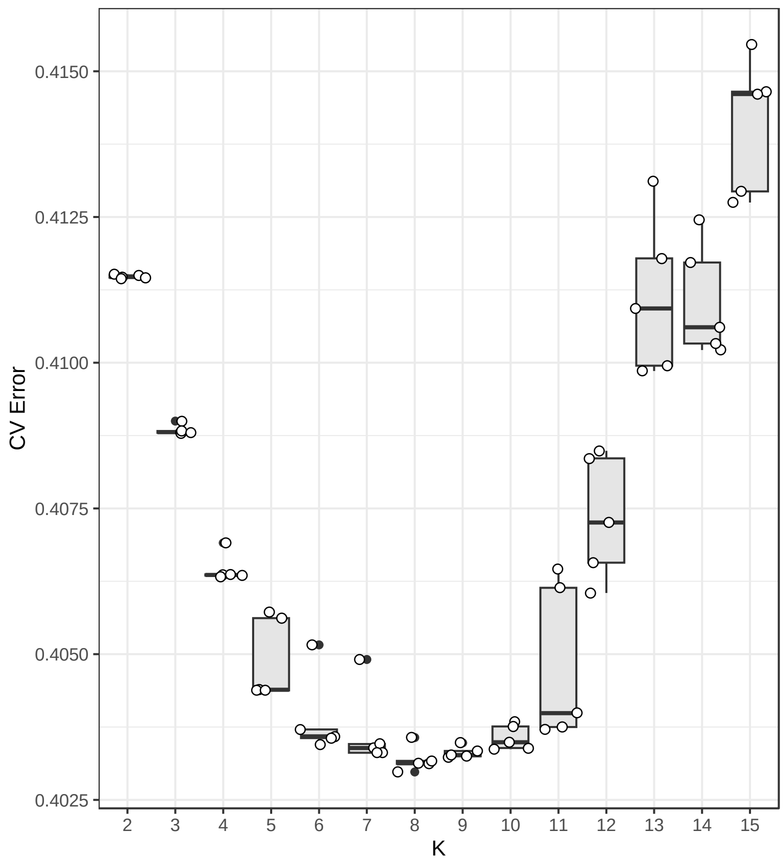

Supplementary Figure 15. Cross-validation errors of ADMIXTURE analysis of present-day and ancient individuals from East and Southeast Asia[44–46,49,50,52–54,59], showing the median of 5 replicates, the 25th and 75th percentiles, and all individual data points, plotted with ggplot2 v3.4.4[62]. Source data are provided as a Source Data file.

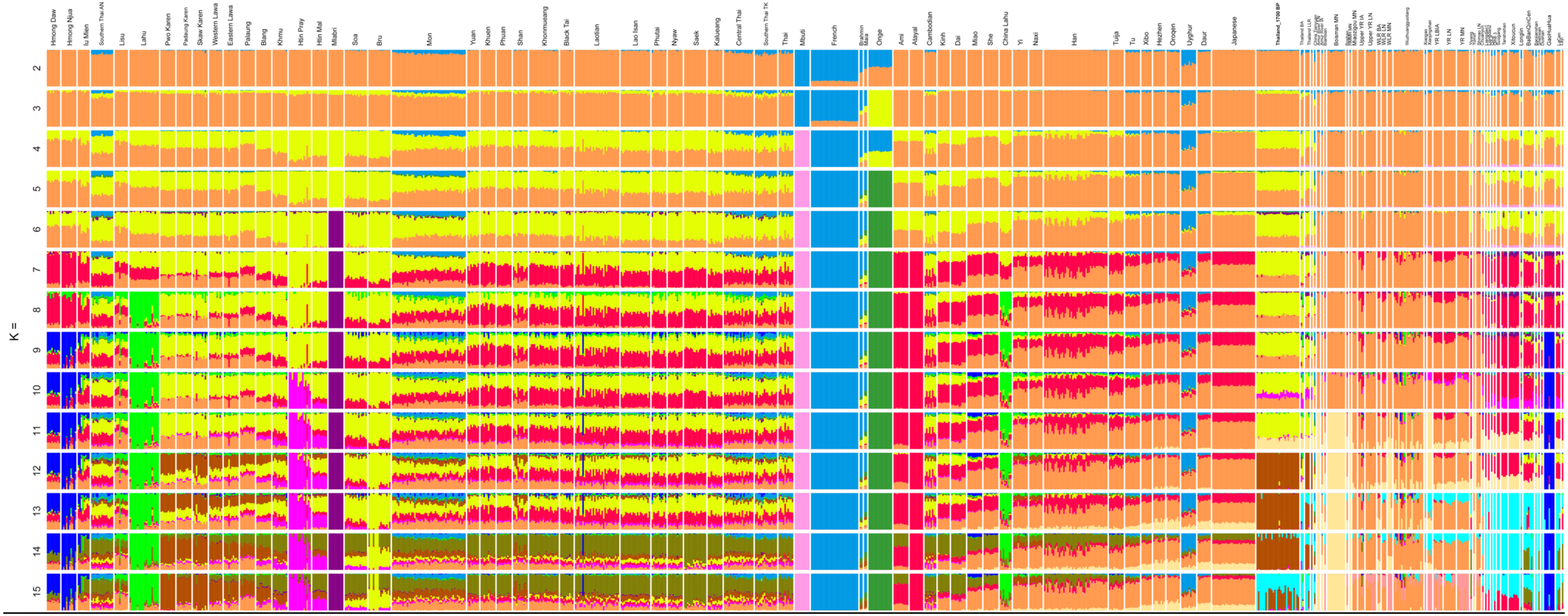

Supplementary Figure 16. ADMIXTURE results of present-day and ancient individuals from East and Southeast Asia[44–46,49,50,52–54,59]; plotted with AdmixturePlotter.R (https://github.com/TCLamnidis/AdmixturePlotter). Source data are provided as a Source Data file.

Supplementary References

1. Treerayapiwat, C. Patterns of Habitation and Burial Activity in the Ban Rai Rock Shelter, Northwestern Thailand. *Asian Perspectives* **44**, 231–245 (2005).
2. Shoocongdej, R. *The Final Report of Survey and Database of Caves in Pang Mapha district, Mae Hong Son Province*. (2000).
3. Shoocongdej, R. *Progress Report 1. Highland Archaeology Project in Pang Mapha Phase I.* (2001).
4. Shoocongdej, R. *Progress Report 2. Highland Archaeology Project in Pang Mapha Phase I.* (2002).
5. Shoocongdej, R. *Progress Report 3. Highland Archaeology Project in Pang Mapha Phase I.* (2002).
6. Shoocongdej, R. *Progress Report 4. Highland Archaeology Project in Pang Mapha Phase I.* (2002).
7. Shoocongdej, R. *Final Report 1. Highland Archaeology Project in Pang Mapha Phase I.* (2003).
8. Shoocongdej, R. *Highland Archaeology in Pang Mapha*. (Silpakorn University Press, 2004).
9. Shoocongdej, R. Late pleistocene activities at the Tham Lod rockshelter in highland Pang Mapha, Mae hong Son province, northwestern Thailand. in *Uncovering Southeast Asia's Past: Selected Papers from the 10th International Conference of the European Association of Southeast Asian Archaeologists* 22–37 (NUS press Singapore, 2006).
10. Marwick, B. Human behavioural ecology and stone artefacts in northwest Thailand during the terminal Pleistocene and Holocene. *Southeast Asian Prehistory: The 11th EurASEAA Conference* 73–80 (2008).

11. Marwick, B. Multiple Optima in Hoabinhian flaked stone artefact palaeoeconomics and palaeoecology at two archaeological sites in Northwest Thailand. *Journal of Anthropological Archaeology* **32**, 553–564 (2013).
12. Shoocongdej, R. & Wattanapituksakul, A. Faunal assemblages and demography during the Late Pleistocene (MIS 2-1) to Early Holocene in Highland Pang Mapha, Northwest Thailand. *Quat. Int.* **563**, 51–63 (2020).
13. Shoocongdej, R. *et al. Final Report of Prehistoric Population and Cultural Dynamic in Highland Pang Mapha, Mae Hong Son Province. Volume II: Archaeology*. (2021).
14. Wannasri, S., Pumijumnong, N. & Shoocongdej, R. Teak log coffin head styles in northern Thailand: Time sequencing with dendrochronology. *Sci. Asia.* **33**, 047 (2007).
15. Pumijumnong, N. & Wannasri, S. Teak Log Coffins in Northwest Thailand: Dated by Dendrochronology and 14Cwiggle Matching. *Applied Environmental Research* **37**, 1–16 (2015).
16. Preechamart, S. *et al.* Tree-ring oxygen isotope chronology of teak log coffins in northwestern Thailand and its relationship with Pacific Decadal Oscillation and El Niño-Southern Oscillation. *Quat. Int.* (2021) doi:10.1016/j.quaint.2021.04.007.
17. Grave, P. Dating of Log Coffins in Northwestern Thailand. (1994).
18. Dilokwanich, S. *An Exploration and Database System of the Caves, Mae Hong Son Province*. (2000).
19. Marwick, B. What attributes are important for the measurement of assemblage reduction intensity? Results from an experimental stone artefact assemblage with relevance to the Hoabinhian of mainland Southeast Asia. *J. Archaeol. Sci.* **35**, 1189–1200 (2008).
20. Chitkament, T., Gaillard, C. & Shoocongdej, R. Tham Lod rockshelter (Pang Mapha district, north-western Thailand): Evolution of the lithic assemblages during the late Pleistocene. *Quat. Int.* **416**, 151–161 (2016).

21. Shoocongdej, R. *Final Report 2. Highland Archaeological Project in Pang Mapha Phase II.* (2007).
22. Suraprasit, K., Shoocongdej, R., Chintakanon, K. & Bocherens, H. Late Pleistocene human paleoecology in the highland savanna ecosystem of mainland Southeast Asia. *Sci. Rep.* **11**, 16756 (2021).
23. Khaewkamput, V. Archaeology of Tham Lod: Late Pleistocene-Late Holocene life ways. in *A Conference Proceeding on Human, Culture, and Paleoenvironment on Highland Pang Pang Mapha, Mae Hong Song Province* (Silpakorn University, Bangkok, 2003).
24. Wattanapituksakul, S. Late Pleistocene Mammal Teeth from the Tham Lod Rockshelter, Amphoe Pang Mapha, Changwat Mae Hong Son. (Chulalongkorn University, Bangkok, 2006).
25. Pautreau, J.-P., Mornais, P. & Doy-Asa, T. *Ban Wang Hai: excavations of an Iron-Age cemetery in northern Thailand*. (Silkworm Books, 2003).
26. Wannasri, S. *A Dendroarchaeological Study of Log Coffins: Bo Krai Cave and Ban Rai Rockshelter in Pang Mapha District, Mae Hong Son Province*. (Mahidol University, 2004).
27. Fellows Yates, J. A. *et al.* Reproducible, portable, and efficient ancient genome reconstruction with nf-core/eager. *PeerJ* **9**, e10947 (2021).
28. Di Tommaso, P. *et al.* Nextflow enables reproducible computational workflows. *Nat. Biotechnol.* **35**, 316–319 (2017).
29. Andrews, S. FastQC A Quality Control tool for High Throughput Sequence Data. https://www.bioinformatics.babraham.ac.uk/projects/fastqc/.
30. Chen, S., Zhou, Y., Chen, Y. & Gu, J. fastp: an ultra-fast all-in-one FASTQ preprocessor. *Bioinformatics* **34**, i884–i890 (2018).
31. Schubert, M., Lindgreen, S. & Orlando, L. AdapterRemoval v2: rapid adapter trimming, identification, and read merging. *BMC Res. Notes* **9**, 88 (2016).

32. Li, H. & Durbin, R. Fast and accurate short read alignment with Burrows-Wheeler transform. *Bioinformatics* **25**, 1754–1760 (2009).

33. Li, H. Aligning sequence reads, clone sequences and assembly contigs with BWA-MEM. *arXiv [q-bio.GN]* (2013).

34. Li, H. *et al.* The Sequence Alignment/Map format and SAMtools. *Bioinformatics* **25**, 2078–2079 (2009).

35. Okonechnikov, K., Conesa, A. & García-Alcalde, F. Qualimap 2: advanced multi-sample quality control for high-throughput sequencing data. *Bioinformatics* **32**, 292–294 (2016).

36. Quinlan, A. R. & Hall, I. M. BEDTools: a flexible suite of utilities for comparing genomic features. *Bioinformatics* **26**, 841–842 (2010).

37. Neukamm, J., Peltzer, A. & Nieselt, K. DamageProfiler: Fast damage pattern calculation for ancient DNA. *Bioinformatics* (2021) doi:10.1093/bioinformatics/btab190.

38. Ewels, P., Magnusson, M., Lundin, S. & Käller, M. MultiQC: summarize analysis results for multiple tools and samples in a single report. *Bioinformatics* **32**, 3047–3048 (2016).

39. Lamnidis, T. C. *et al.* Ancient Fennoscandian genomes reveal origin and spread of Siberian ancestry in Europe. *Nat. Commun.* **9**, 5018 (2018).

40. Korneliussen, T. S., Albrechtsen, A. & Nielsen, R. ANGSD: Analysis of Next Generation Sequencing Data. *BMC Bioinformatics* **15**, 356 (2014).

41. Jun, G., Wing, M. K., Abecasis, G. R. & Kang, H. M. An efficient and scalable analysis framework for variant extraction and refinement from population-scale DNA sequence data. *Genome Res.* **25**, 918–925 (2015).

42. Sawyer, S., Krause, J., Guschanski, K., Savolainen, V. & Pääbo, S. Temporal patterns of nucleotide misincorporations and DNA fragmentation in ancient DNA. *PLoS One* **7**, e34131 (2012).

43. Ringbauer, H., Novembre, J. & Steinrücken, M. Parental relatedness through time revealed by runs of homozygosity in ancient DNA. *Nat. Commun.* **12**, 5425 (2021).

44. McColl, H. *et al.* The prehistoric peopling of Southeast Asia. *Science* **361**, 88–92 (2018).

45. Patterson, N. *et al.* Ancient admixture in human history. *Genetics* **192**, 1065–1093 (2012).

46. Lazaridis, I. *et al.* Ancient human genomes suggest three ancestral populations for present-day Europeans. *Nature* **513**, 409–413 (2014).

47. Jeong, C. *et al.* Long-term genetic stability and a high-altitude East Asian origin for the peoples of the high valleys of the Himalayan arc. *Proc. Natl. Acad. Sci. U. S. A.* **113**, 7485–7490 (2016).

48. Skoglund, P. *et al.* Genomic insights into the peopling of the Southwest Pacific. *Nature* **538**, 510–513 (2016).

49. Lipson, M. *et al.* Ancient genomes document multiple waves of migration in Southeast Asian prehistory. *Science* **361**, 92–95 (2018).

50. Ning, C. *et al.* Ancient genomes from northern China suggest links between subsistence changes and human migration. *Nat. Commun.* **11**, 2700 (2020).

51. Yang, M. A. *et al.* 40,000-Year-Old Individual from Asia Provides Insight into Early Population Structure in Eurasia. *Curr. Biol.* **27**, 3202-3208.e9 (2017).

52. Yang, M. A. *et al.* Ancient DNA indicates human population shifts and admixture in northern and southern China. *Science* **369**, 282–288 (2020).

53. Kutanan, W. *et al.* Reconstructing the Human Genetic History of Mainland Southeast Asia: Insights from Genome-Wide Data from Thailand and Laos. *Mol. Biol. Evol.* **38**, 3459–3477 (2021).

54. Wang, T. *et al.* Human population history at the crossroads of East and Southeast Asia since 11,000 years ago. *Cell* (2021) doi:10.1016/j.cell.2021.05.018.

55. Wang, C.-C. *et al.* Genomic insights into the formation of human populations in East Asia. *Nature* **591**, 413–419 (2021).

56. Patterson, N., Price, A. L. & Reich, D. Population structure and eigenanalysis. *PLoS Genet.* **2**, e190 (2006).

57. Pittayaporn, P. Layers of Chinese Loanwords in Protosouthwestern Tai as Evidence for the Dating of the Spread of Southwestern Tai. *Manusya: Journal of Humanities* **17**, 47–68 (2014).

58. Alexander, D. H., Novembre, J. & Lange, K. Fast model-based estimation of ancestry in unrelated individuals. *Genome Res.* **19**, 1655–1664 (2009).

59. Mallick, S. *et al.* The Simons Genome Diversity Project: 300 genomes from 142 diverse populations. *Nature* **538**, 201–206 (2016).

60. Narasimhan, V. M. *et al.* The formation of human populations in South and Central Asia. *Science* **365**, (2019).

61. Kahle, D. & Wickham, H. ggmap: Spatial visualization with ggplot2. *The R Journal* **5**, 144–161 (2013).

62. Wickham, H. *Ggplot2*. (Springer International Publishing, 2016).
