# Supplementary material for: Genomic portrait and relatedness patterns of the Iron Age Log Coffin culture in northwestern Thailand
Source: Nat Commun. 2023 Dec 22;14:8527. doi: 10.1038/s41467-023-44328-2 (PMC10746721; doi:10.1038/s41467-023-44328-2)
Supplement: Supplementary file 3 — Description of Additional Supplementary Files [file 41467_2023_44328_MOESM3_ESM.pdf]

Description of Additional Supplementary Files

Supplementary Data 1. Archaeological and genomic information of Iron Age Log Coffin culture-associated individuals from northwestern Thailand.

Supplementary Data 2. Direct dating results of a selection of petrous bones from Log Coffin sites in Pang Mapha, Thailand as provided by the Klaus-Tschira-Archäometrie-Zentrum, Germany.

Supplementary Data 3. Close genetic relatedness estimation of the Log Coffin-associated individuals. Results of genetic relatedness inference with KIN v.3.1.3, k0 shows the proportion of the genome without IBD sharing, k1 the proportion with one chromosome in IBD, k2 the proportion with both chromosomes in IBD.

Supplementary Data 4. Amount and length of genetic blocks identical-by-descent (IBD) between newly generated Log Coffin-associated individuals summed across all chromosomes.

Supplementary Data 5. Admixture proportion estimations modelling ancient genomes from mainland Southeast Asia as a three-way admixture of hunter-gatherer, Yangtze River- and Yellow River-related ancestry with qpAdm v650. Well-fitting p-values are marked in red, poor-fitting nested p-values and negative admixture proportions are marked in red, standard deviation as calculated with block jackknife in qpAdm v650.
