# Supplementary material for: Genomic portrait and relatedness patterns of the Iron Age Log Coffin culture in northwestern Thailand
Source: Nat Commun. 2023 Dec 22;14:8527. doi: 10.1038/s41467-023-44328-2 (PMC10746721; doi:10.1038/s41467-023-44328-2)
Supplement: Supplementary file 9 — Reporting Summary [file 41467_2023_44328_MOESM9_ESM.pdf]

nature portfolio

Corresponding author(s): Selina Carlhoff, Rasmi Shoocongdej, Johannes Krause

Last updated by author(s): 17/11/23

Reporting Summary

Nature Portfolio wishes to improve the reproducibility of the work that we publish. This form provides structure for consistency and transparency in reporting. For further information on Nature Portfolio policies, see our Editorial Policies and the Editorial Policy Checklist.

Statistics

For all statistical analyses, confirm that the following items are present in the figure legend, table legend, main text, or Methods section.

n/a	Confirmed	
☐	☒	The exact sample size (*n*) for each experimental group/condition, given as a discrete number and unit of measurement
☐	☒	A statement on whether measurements were taken from distinct samples or whether the same sample was measured repeatedly
☐	☒	The statistical test(s) used AND whether they are one- or two-sided<br>*Only common tests should be described solely by name; describe more complex techniques in the Methods section.*
☐	☒	A description of all covariates tested
☐	☒	A description of any assumptions or corrections, such as tests of normality and adjustment for multiple comparisons
☐	☒	A full description of the statistical parameters including central tendency (e.g. means) or other basic estimates (e.g. regression coefficient) AND variation (e.g. standard deviation) or associated estimates of uncertainty (e.g. confidence intervals)
☐	☒	For null hypothesis testing, the test statistic (e.g. *F*, *t*, *r*) with confidence intervals, effect sizes, degrees of freedom and *P* value noted<br>*Give P values as exact values whenever suitable.*
☒	☐	For Bayesian analysis, information on the choice of priors and Markov chain Monte Carlo settings
☒	☐	For hierarchical and complex designs, identification of the appropriate level for tests and full reporting of outcomes
☒	☐	Estimates of effect sizes (e.g. Cohen's *d*, Pearson's *r*), indicating how they were calculated

Our web collection on statistics for biologists contains articles on many of the points above.

Software and code

Policy information about availability of computer code

Data collection	No software was used for data collection.
Data analysis	nf-core/eager v2.4.0 (Nextflow v21.04.0, FastQC v0.11.937, fastP v0.20.1, AdapterRemoval v2.3.2, BWA v0.7.17-r1188, SAMtools v1.12, Qualimap v2.2.2-dev43, bedtools v2.30.0, MarkDuplicates v2.26.0, DamageProfiler v0.4.9, endorS.py v0.4, MultiQC v1.11, MTNucRatioCalculator v0.7, sexdeterrmine v1.1.2, ANGSD v0.935, bamUtil v1.0.15, pileupCaller v1.4.0.5)<br>ContamMix v1.0-10<br>KIN v3.1.3<br>hapROH v0.6, Jupyter notebooks v6.4.10<br>Geneious v2019.2.3, HaploGrep v2.4.0<br>EIGENSOFT v7.2.1 (smartpca v16000)<br>AdmixTools v7.0.2 (qp3Pop v651, qpDstat v980, qpAdm v1520), qpWrapper v1.0.0, qpParser v0.1.1<br>ADMIXTURE v1.3.0<br>ATLAS v.0.9, GLIMPSE v1.0.0, ancIBD v.0.5<br>ggmap v4.0.0, DataGraph v4.4<br>R code for visualisation is available on GitHub

For manuscripts utilizing custom algorithms or software that are central to the research but not yet described in published literature, software must be made available to editors and reviewers. We strongly encourage code deposition in a community repository (e.g. GitHub). See the Nature Portfolio guidelines for submitting code & software for further information.

Data

Policy information about availability of data

All manuscripts must include a data availability statement. This statement should provide the following information, where applicable:

- Accession codes, unique identifiers, or web links for publicly available datasets
- A description of any restrictions on data availability
- For clinical datasets or third party data, please ensure that the statement adheres to our policy

The newly generated raw nuclear sequences are available at the European Nucleotide Archive under the accession number PRJEB59488.
The reference genome is available at the NCBI under accession number GCF_000001405.25. Imputation reference data is available from the 1000 Genomes Project Phase 3. Previously published data used in principal component analysis and admixture modelling is available from the Poseidon Community Archive and at the Genome Sequencing Archive for Human under accession number HRA000451. Source Data for Figures 2c and Supplementary Figures 5B can be found in Supplementary Data 4. Source data for all other figures are provided as a Source Data file.

Human research participants

Policy information about studies involving human research participants and Sex and Gender in Research.

Reporting on sex and gender	In this study, only the karyotypes of sampled individuals were determined to inform X- and Y-chromosome-specific analysis, i.e. nuclear contamination and Y-haplogroup assessment.
Population characteristics	There were no covariate-relevant population characteristics.
Recruitment	Selection of individuals for data generation was only determined by availability of archaeological material relevant for the research questions.
Ethics oversight	The human remains were analysed with permission of the Fine Arts Department of Thailand (permit numbers 497/2561 and WTh-0417-236).

Note that full information on the approval of the study protocol must also be provided in the manuscript.

Field-specific reporting

Please select the one below that is the best fit for your research. If you are not sure, read the appropriate sections before making your selection.

☒ Life sciences ☐ Behavioural & social sciences ☐ Ecological, evolutionary & environmental sciences

For a reference copy of the document with all sections, see nature.com/documents/nr-reporting-summary-flat.pdf

Life sciences study design

All studies must disclose on these points even when the disclosure is negative.

Sample size	No statistical methods were used to determine sample size a priori. Sample size was dependent on the availability of archaeological material relevant for the research questions.
Data exclusions	We only excluded data after initial quality control, if genomic coverage was too low or contamination levels were too high. Genetic first degree relatives were excluded from f-statistics and admixture modeling to avoid biasing the analyses. These are established filtering steps within archaeogenetics.
Replication	Replication of results from archaeological material is challenging, as the sampled individuals are unique. However, we could replicate previously published results of individuals from the same time period and region, who were analysed at a different institution.
Randomization	Since the sampled individuals were not pre-selected or grouped a priori, the archaeological individuals were inherently random.
Blinding	Blinding was not relevant for this study, as individuals were grouped based on archaeological information.

Reporting for specific materials, systems and methods

We require information from authors about some types of materials, experimental systems and methods used in many studies. Here, indicate whether each material, system or method listed is relevant to your study. If you are not sure if a list item applies to your research, read the appropriate section before selecting a response.

Materials & experimental systems

n/a	Involved in the study
☒	☐ Antibodies
☒	☐ Eukaryotic cell lines
☐	☒ Palaeontology and archaeology
☒	☐ Animals and other organisms
☒	☐ Clinical data
☒	☐ Dual use research of concern

Methods

n/a	Involved in the study
☒	☐ ChIP-seq
☒	☐ Flow cytometry
☒	☐ MRI-based neuroimaging

Palaeontology and Archaeology

Specimen provenance

The sampled individuals were excavated at eight archaeological sites in Mae Hong Son and Lamphun Province, Thailand: Ban Rai (n=5), Lahu Pot (n=2), Moa Mussur (n=4), Pang Kham 1 (n=1), Tham Lod (n=6), Wang Hai (n=2), Yappa Nhae 1 (n=6) and Yappa Nhae 2 (n=35). The sites are described in detail in the supplementary information. The excavations were conducted with permission of the Fine Arts Department of Thailand (permit numbers 497/2561 and WTh-0417-236).

Specimen deposition

The sampled skeletal remains are curated by Prof. Dr. Rasmi Shoocongdej at Silpakorn University, Bangkok, Thailand.

Dating methods

Ten petrous bones were dated through Accelarator Mass Spectrometry at the Klaus-Tschira-Archäometrie-Zentrum, Mannheim, Germany (MAMS). Collagen was extracted from the bone samples (modified Longin method), purified by ultrafiltration (fraction >30kD) and freeze-dried. 14C was analyzed using a MICADAS-type AMS system, where the isotopic ratios 14C/12C and 13C/12C of samples, calibration standard (Oxalic Acid-II), blanks and control standards are measured simultaneously. 14C-ages were normalized to delta13C=-25‰ (Stuiver & Polach, 1977) and calibrated using the dataset INTCAL13 and software SwissCal (L.Wacker, ETH-Zürich). Calibration graphs were generated using the software OxCal v4.3.2.

☒ Tick this box to confirm that the raw and calibrated dates are available in the paper or in Supplementary Information.

Ethics oversight

The human remains were excavated, exported, sampled for ancient DNA, and analysed with permission of the Fine Arts Department of Thailand (permit numbers 497/2561 and WTh-0417-236).

Note that full information on the approval of the study protocol must also be provided in the manuscript.
